# Novel nastorazepide-based radioligands demonstrate effective cholecystokinin-2 receptor targeting with potential for theranostic applications

**DOI:** 10.7150/thno.129327

**Published:** 2026-07-29

**Authors:** Lennart Bohrmann, Doroteja Novak, Marko Kroselj, Raghuvir H. Gaonkar, Francesca Porto, Melpomeni Fani, Petra Kolenc, Marko Anderluh, Rosalba Mansi

**Affiliations:** 1 Division of Radiopharmaceutical Chemistry, University Hospital Basel, Petersgraben 4, 4031 Basel, Switzerland.; 2 Division of Nuclear Medicine, University Medical Centre Ljubljana, Zaloška 7, 1000 Ljubljana, Slovenia.; 3 Department of Pharmaceutical Chemistry, Faculty of Pharmacy, University of Ljubljana, Aškerčeva 7, 1000 Ljubljana, Slovenia.

**Keywords:** cholecystokinin-2 receptor (CCK2R) antagonists, nastorazepide, radioligands, SPECT/CT, peptide receptor radionuclide therapy

## Abstract

Radiolabeled gastrin antagonists are emerging as a new tool for the diagnosis and treatment of cholecystokinin-2 receptor (CCK2R) expressing tumors such as medullary thyroid carcinoma and small cell lung cancer, overcoming limitations of agonist-based ligands. In this study, the CCK2R antagonist nastorazepide (Z-360) was used as a targeting scaffold in combination with either a peptidic (NPP) or a hydrophilic polysaccharide (NPS) linker and the chelators DOTA and DOTAGA, yielding four novel radioligands. These were evaluated alongside the reference minigastrin analogs DOTA-PP-F11 and DOTA-MGS5.

**Methods:**

All ligands were synthetized *via* solid-phase peptide synthesis and labeled with In-111 and Lu-177. *In vitro* characterization included receptor affinity, hydrophilicity and cellular uptake in A431-CCK2R. *In vivo* biodistribution and imaging were performed in A431-CCK2R xenografts. [^177^Lu]Lu-DOTAGA-NPP was further assessed in a proof-of-concept therapy study and metabolic stability assays.

**Results:**

^111^In-labeled antagonists showed 3-4-fold higher site recognition than [^111^In]In-DOTA-PP-F11 and nanomolar affinities comparable to the reference ligand. Among the tested ligands, [^111^In]In-DOTAGA-NPP demonstrated the highest cellular uptake (22.0 ± 0.2%), approximately twice that of the other ligands and moderately higher than [^111^In]In-DOTA-PP-F11 (16.2 ± 1.8%) after 4 h at 37 °C. *In vivo*, all ^177^Lu-labeled antagonists demonstrated high tumor uptake (range: 22.98 ± 7.04 - 29.00 ± 3.30% I.A./g tissue) with [^177^Lu]Lu-DOTAGA-NPP exhibiting the most favorable tumor-to-tissue ratios. Compared with [^177^Lu]Lu-DOTA-MGS5, [^177^Lu]Lu-DOTAGA-NPP showed rapid tumor accumulation with faster washout but reduced stomach exposure and high *in vivo* stability.

**Conclusion:**

[¹⁷⁷Lu]Lu-DOTAGA-NPP emerged as the most promising lead compound, combining high tumor uptake, favorable tumor-to-organ ratios, and excellent *in vivo* stability. Although no significant therapeutic efficacy was observed in the tested xenograft model, the results support [^177^Lu]Lu-DOTAGA-NPP as a promising lead for optimization. Overall, these findings highlight the potential of Z-360-based CCK2R antagonists as scaffolds for next-generation theranostic radioligands and support their development as alternatives to minigastrin-based tracers.

## Introduction

The clinical success of radiolabeled somatostatin analogs has driven research in developing peptide radioligands targeting different receptors overexpressed in various human tumors. The cholecystokinin-2 receptor (CCK2R) stands out as a highly promising target for the development of targeted radioligands due to its high expression in several tumor entities, including medullary thyroid carcinomas (MTC) (92%), small cell lung cancers (SCLC) (57%), astrocytomas (65%), and stromal ovarian cancers (100%) 1, 2. Many radioligands targeting CCK2R have been developed, most based on peptide analogs of the natural ligand minigastrin 3. Among these, the minigastrin derivatives DOTA-PP-F11 (DOTA-(D-Glu)₆-Ala-Tyr-Gly-Trp-Met-Asp-Phe-NH₂) and its methionine-free analog DOTA-PP-F11N are the most clinically advanced 4, 5. A phase 0 study involving patients with metastatic MTC confirmed [¹⁷⁷Lu]Lu-DOTA-PP-F11N accumulation at tumor sites in all participants. However, rapid *in vivo* degradation of the radioligands led to relatively low radiation doses delivered to metastases, limiting their therapeutic efficacy 6.

Efforts to enhance stability have led to the development of improved compounds such as MGS5 7 and CCK-66 8. In particular, [^68^Ga]Ga-DOTA-MGS5, demonstrated high diagnostic accuracy in PET/CT imaging of advanced MTC and SCLC, along with favorable safety and dosimetry profiles in a phase I/IIA trial 9. Despite these advances, the agonistic nature of these ligands may limit their therapeutic application, as receptor activation can induce dose-dependent side effects such as nausea, hypotension, and dizziness at therapeutic doses due to receptor activation 5, 6, 10. Stomach uptake is also a major dose-limiting factor because physiologic CCK2R expression in gastric tissue leads to relevant off-target accumulation, thereby narrowing the therapeutic window 5. In this context, CCK2R antagonists could potentially mitigate these side effects during receptor-targeted radioligand therapy (RLT) while preserving tumor targeting and improving the tumor-to-normal-organ ratio. Moreover, antagonists could offer additional advantages, including higher binding capacity and greater tumor accumulation, as seen with other G protein-coupled receptor (GPCR) antagonists compared to agonists such as somatostatin and bombesin based antagonists 11, 12.

Z-360 (nastorazepide), a benzodiazepine derivative (3-[[1-cyclohexyl-5-(3,3-dimethyl-2-oxobutyl)-4-oxo-2,3-dihydro-1,5-benzo-diazepin-3-yl]-carbamoylamino -benzoic acid), is a non-peptidic CCK2R antagonist with high affinity to the human receptor, able to effectively block gastrin or CCK-mediated receptor activation. Extensive preclinical work has shown that Z-360 inhibits tumor growth in gastrin responsive cancers 13, 14. Early phase clinical trials in advanced pancreatic cancer patients demonstrated that Z-360 is safe and well tolerated when combined with gemcitabine, with nearly 50% of patients reporting clinically relevant pain relief 15. Importantly, Z-360 contains an accessible free carboxylic acid group, providing a versatile site for conjugation with a wide range of functional moieties such as cytotoxic agents, fluorescent dyes, or metal chelates 16-18. Recently, radioligands derived from Z-360, functionalized with various chelators and radiometals have been developed 19-23. Their preclinical evaluation revealed several key advantages: Z-360-based radioligands offer high metabolic stability, improved pharmacokinetics with low stomach uptake (a CCK2-positive organ), rapid clearance from non-target tissues and, most importantly, preventing receptor activation. However, these studies also showed that the choice of linker and chelator is essential to achieve high affinity and optimal biodistribution profile of these ligands. We developed novel radioligands by exploring different combinations of linkers and chelators aiming for optimal *in vivo* properties for theranostic applications. We investigated two linker systems previously reported by Wayua *et al*. 19, which differ in their chemical and pharmacokinetic properties. The peptidic NPP linker consists of the tripeptide Glu-Arg-DAsp and was rationally designed to increase hydrophilicity and overall polarity through a balanced combination of acidic (Glu, Asp) and basic (Arg) residues, thereby improving background clearance while preserving receptor affinity. In contrast, the peptidosaccharide NPS linker was introduced to minimize nonspecific kidney and liver uptake by reducing recognition by scavenger receptors, which are known to bind and internalize peptidic conjugates. In this study, four novel CCK2R antagonistic radioligands were generated combining Z-360 with the NPP or the NPS linker and the bifunctional chelators DOTA and DOTAGA. These candidates were evaluated preclinically against the established agonists DOTA-PP-F11 and DOTA-MGS5 to identify the most promising compound for theranostic use.

## Materials and Methods

### General information

All materials and additional methods are provided in the Supplementary Information.

### Synthesis

**Figure 1:**
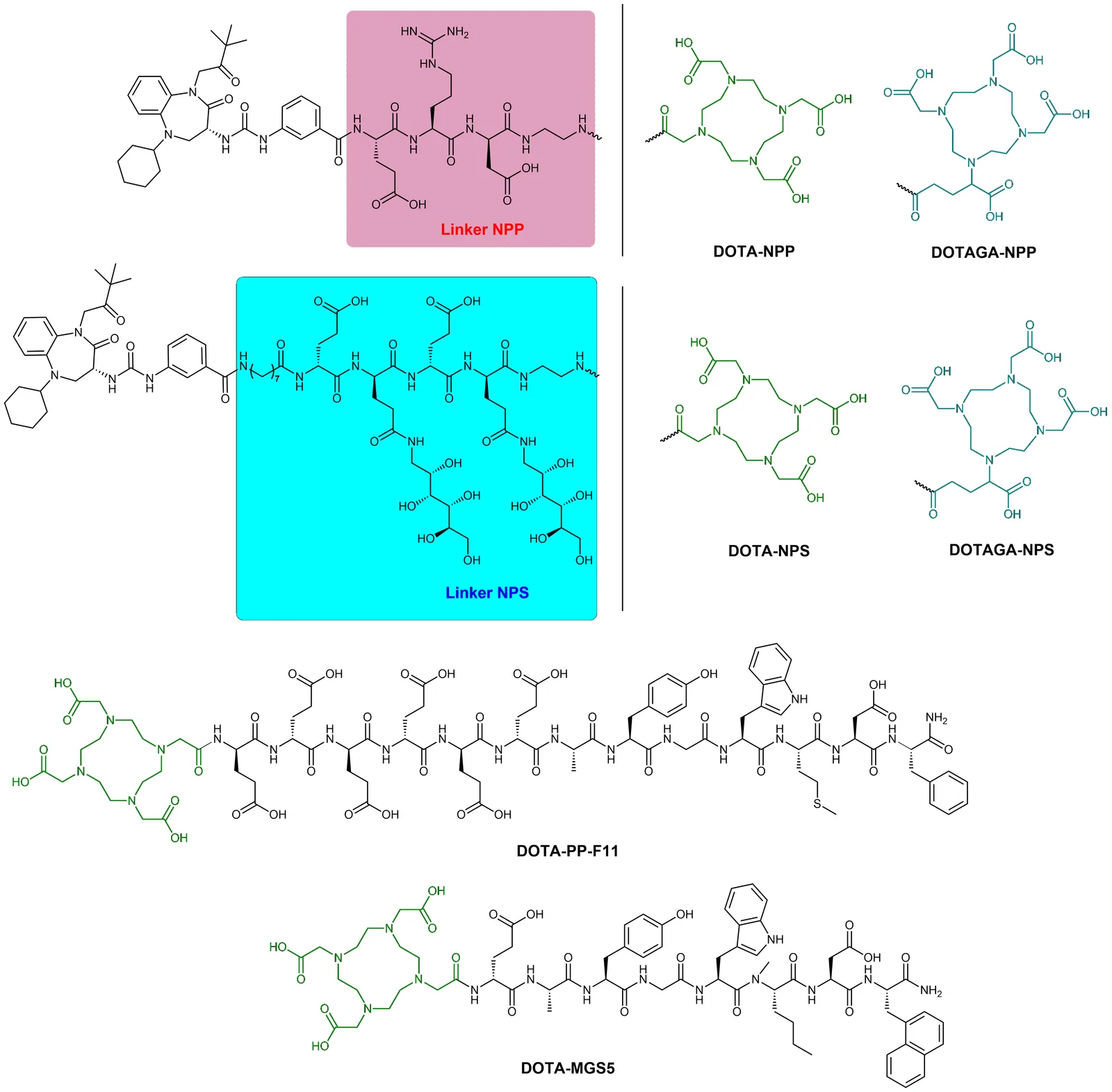
Chemical structures of the studied ligands: DOTA-NPP, DOTAGA-NPP, DOTA-NPS, DOTAGA-NPS, DOTA-PP-F11 and DOTA-MGS5.

Z-360 was used as vector moiety and combined with the bifunctional chelators DOTA and DOTAGA *via* two different spacers to obtain the four CCK2R binding ligands, Z-360-Glu-Arg-DAsp-DOTA (DOTA-NPP), Z-360-Glu-Arg-DAsp-DOTAGA (DOTAGA-NPP), Z-360-8Aoc-DGlu-PS-DGlu-PS-DOTA (DOTA-NPS) and Z-360-8Aoc-DGlu-PS-DGlu-PS-DOTAGA (DOTAGA-NPS), (where 8Aoc is 8-amino-octanoic acid and PS is 3,4,5,6-di-O-isopropylidene-1-amino-1-deoxy-D-glucitol-γ-glutamate) (Figure 1). The peptides were synthesized on solid-phase following the Fmoc/tBu strategy using the acid-sensitive 2-chlorotryl chloride resin, chelators were coupled in solution as detailed in the Supplementary Information. The final ligands were purified by semi-preparative RP-HPLC and characterized by RP-HPLC and ESI-MS.

DOTA-PP-F11 and DOTA-MGS5 were synthesized by standard solid phase peptide synthesis on Rink amide MBHA resin according to published procedures 24, 25.

### Radiolabeling and *in vitro* characterization

Stock solutions. Ligands were prepared as stock solutions in trace selected water at concentrations of approximately 1 μg/μL (500–700 μM) and radiolabeled with ¹¹¹In and ¹⁷⁷Lu as described in the protocols below.

Radiolabeling ***with indium-111.*** Each ligand (5–7 nmol) was mixed with [^111^In]InCl_3_ (50–150 MBq) in MES buffer (0.5 M, pH 5.5) and heated at 95 °C for 30 min to yield the corresponding ¹¹¹In-labeled radioligand. A small aliquot was withdrawn from the radiolabeled solution, added to Ca-DTPA (0.1 M, 50 µL) and injected into the radio-HPLC for quality control. The labeled solutions were diluted with 0.9% NaCl to obtain the desired dilutions depending on the planned experiments.

***Radiolabeling with lutetium-177.*** Each ligand was combined with varying activity of [^177^Lu]LuCl_3_ in ammonium acetate buffer (0.4 M, pH 5.0, 250 μL) and heated at 95 °C for 30 min to yield the corresponding ¹⁷⁷Lu-labeled radioligand. A maximum specific activity of 200 MBq/nmol was achieved for therapy experiments, while lower specific activity was used for all the other experiments. A small aliquot was withdrawn from the radiolabeled solution, added to Ca-DTPA (0.1 M, 50 µL) and injected on the radio-HPLC for quality control. The labeled solutions were diluted with 0.9% NaCl to obtain the final dilution for further experiments.

log D (distribution coefficient) determination. For log *D* determination, the radiolabeled compound (approx. 0.3 MBq/µL, 10 µL, 200 pmol) was added to a biphasic system of equal volumes of PBS (pH 7.4) and n-octanol (500 µL each). After vortexing and shaking for 30 min, phases were separated by centrifugation (5,000 g, 10 min). Radioactivity was quantified in triplicate 100 µL aliquots from each phase using a γ-counter. The distribution coefficient was determined as the logarithmic ratio of activity in the aqueous phase and organic fraction (log D_pH=7.4_ = log10 (counts octanol phase / counts aqueous phase).

Serum protein binding. Serum protein binding was determined using fresh human serum. The detailed procedure is described in the Supplementary Information.

### *In vitro* experiments

Cell line. The human epidermoid carcinoma cell line (A431) stably transfected with CCK2R (A431-CCK2R) and the mock cell line (A431-mock) were used. Both cell lines were a kind gift of Prof. Clemens Decristoforo (Medical University Innsbruck, Innsbruck, Austria), originally generated by Prof. Luigi Aloj (University of Cambridge, UK) 26**.** Cells were maintained under standard conditions (37ºC, 5% CO_2_ in humidified atmosphere) in Dulbecco’s modified Eagle’s medium (DMEM; Gibco Thermo Fischer Scientific, USA) supplemented with 10% (v/v) fetal calf serum (Gibco, Thermo Fischer Scientific, USA), 250 μg/mL G418 (Merck, Germany), 100 U/mL penicillin and 100 μg/mL streptomycin (Sigma-Aldrich, USA). Cells were cultured to 80% confluence before being harvested with 0.25% EDTA-trypsin solution (Gibco, Thermo Fischer Scientific, USA), the day prior the experiment (1x10^6^ cells/well) and incubated overnight at 37 °C, 5% CO_2_. On the day of the experiment, the medium was replaced by fresh medium supplemented with 1% FBS and incubated for 1 h at 37 °C, 5% CO_2_.

***Determination of antagonistic properties.*** Half maximal inhibitory concentration (IC_50_) was assessed by measuring IP1 accumulation using the Cisbio IPOne^®^ HTRF assay (Cisbio Perkin Elmer, USA). A431-CCK2R cells were stimulated with gastrin-17 (G17) as agonist at its previously determined EC_90_ concentration 22, and IC_50_ values were derived from concentration-response curves. The detailed procedure is provided in the supplementary information.

Cell-association studies. ^111^In-labeled ligands (1 pmol, final concentration in well: 0.667 nM) were added to the plates and incubated for 0.5, 1, 2 and 4 h at 37 °C, 5% CO_2_. Non-specific binding was determined using mock A431 cells or adding 1000-fold excess of the blocking agents on selected A431-CCK2R positive cells. At each time point, the cell plates were transferred to ice to stop the internalization. Then, the medium was removed, the cells were washed twice with ice-cold PBS and collected as free fraction. Afterwards, surface-bound radioligand was removed by washing two times with ice-cold glycine buffer (0.5 M, pH 2.8, 5 min each), and the combined washes collected as surface-bound fraction. Cells were subsequently lysed with 1M NaOH to recover the internalized fraction.

The radioactivity in all fractions was counted in γ-counter. The cell associated fraction accounts for the sum of internalized and bound fraction. Specific cellular uptake has been determined as the difference between the total and the non-specific cellular uptake. The experiments were carried out in triplicates.

Saturation binding studies. The cells were kept at room temperature for half an hour before the start of the experiment. ^111^In-labeled ligands were added in increasing concentrations (final concentration range: 0.03–500 nM) and incubated for 2 h at room temperature. After incubation, the medium was removed, the cells were washed with ice-cold PBS and the supernatants combined as free fraction. Subsequently, the cells were lysed with 1 M NaOH and the lysate was collected as cell associated fraction. The radioactivity in all fractions was then counted in γ-counter. Nonspecific binding was assessed using A431-mock cells treated according to the procedure outlined above. The K_D_ and B_max_ were subsequently derived usingGraphPad Prism (version 10.1.2, GraphPad Software, USA). All experiments were performed in triplicate.

### *In vivo* studies

Animals. The animal experiments were approved and carried out in accordance with the Swiss regulations for animal treatment (approval no. 2799). Four to six weeks-old female, athymic nude mice (Foxn1^nu^, Envigo, The Netherlands) were subcutaneously inoculated with A431-CCK2R cells (1×10^7^ cells, suspended in 100 μL sterile PBS) on the shoulder for biodistribution and SPECT/CT imaging. The tumors were then allowed to grow for approximately 7–10 days until they reached a size of 100–200 mm^3^. The primary goal of the study was the relative comparison of radioligands and no relevant sex-dependent effects were anticipated. Biodistribution studies were performed only in female mice to maintain experimental consistency.

Biodistribution. The radioligands [^177^Lu]Lu-DOTA-PP-F11, [^177^Lu]Lu-DOTA-MGS5, [^177^Lu]Lu-DOTA-NPP, [^177^Lu]Lu-DOTAGA-NPP, [^177^Lu]Lu-DOTA-NPS or [^177^Lu]Lu-DOTAGA-NPS (20 pmol/0.4–1.0 MBq, n = 3–8 per group) were injected in CCK2R xenografts and biodistribution studies performed at different time points (between 1 h and 130 h post injection (p.i.)). At the time of sacrifice the tumor weights ranged between 0.2 g and 1.2 g. Organs of interest were harvested, emptied where relevant (stomach, intestine, and heart), blotted dry, weighed, and assayed in a gamma counter. The data are reported as percentage of injected activity per gram (% IA/g), determined by extrapolating from the counts of a standard aliquot drawn from the injected solution. Multiple unpaired t-testing using Holm-Šidák correction was used to determine statistical differences between the compounds at each time point.

To calculate the AUC, decay-corrected time-activity data were fitted using a mono-exponential function in GraphPad Prism to estimate the initial activity concentration (Y_0_) and the biological clearance rate constant λ_biol_. The physical decay constant of ^177^Lu was calculated from its half-life and the effective clearance constant for each radioligand was defined as λ_eff =_ λ_biol. +_ λ_phys_. The AUC was calculated analytically as AUC = Y_0_/ λ_eff_ and expressed in units of %IA/g × h. Uncertainty was estimated by propagation of the standard error of the fitted parameters Y_0_ and λ_biol._ obtained from nonlinear regression. Blocking studies were performed with [^177^Lu]Lu-DOTAGA-NPP, by 5 min pre-injection of the corresponding unlabeled compound in 1’000-times molar excess (20 nmol), at both 1 h and 4 h post-injection.

***Dosimetry****.* Extrapolation of mouse biodistribution data to human absorbed doses was based on interspecies scaling using the organ and body weight from male and female phantoms in OLINDA dosimetry software (version 1.0, Vanderbilt University), according to the formula below:



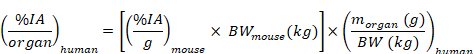



where BW is the body weight and morgan the organ mass. Allometric scaling was further applied to account for the more rapid pharmacokinetics observed in mice relative to humans, enabling human residence times to be estimated:



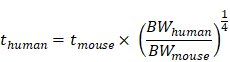



assuming a body weight of 25 g for the mice. The extrapolated data were entered into OLINDA to compute the human absorbed dose per unit of administered activity. Calculations were performed using the adult female and male reference phantoms.

SPECT/CT imaging ***study***. SPECT/CT images were acquired for [^177^Lu]Lu-DOTAGA-NPP (100 µL, 200 pmol/5-6 MBq) at 4 h and 24 h p.i. Mice were imaged supine, head first, using a SPECT/CT system dedicated to imaging small animals (NanoSPECT/CTTM Bioscan, Mediso Medical Imaging Systems, Budapest, Hungary). SPECT/CT scanners details are provided in Supplementary Information.

***In vivo metabolic stability*.** The *in vivo* stability was assessed in healthy BALB/c mice after intravenous injection of [^177^Lu]Lu-DOTAGA-NPP, [^177^Lu]Lu-DOTA-PP-F11 and [^177^Lu]Lu-DOTA-MGS5 (100 μL/500 pmol/25 MBq). The mice were euthanized at 30 min and 60 min p.i. by CO_2_, blood and urine samples were collected in polypropylene tubes containing EDTA. The mixtures were vortexed and subsequently centrifuged at 4,000 g for 10 min at 4 °C. Kidneys were quickly removed after euthanasia and approximately 100 mg specimens were transferred to ice-cooled polypropylene tubes containing 800 µL 1% ReadyShield® Protease Inhibitor Cocktail (Sigma-Aldrich) in PBS. The tissues were homogenized using an Ultra-Turrax homogenizer, followed by centrifugation at 15,000 g for 15 min at 4 °C. The supernatant of each sample was collected, mixed with methanol (v/v 1:2) and centrifuged again for additional 15 min. Aliquots of the supernatant were diluted 1:1 with H_2_O up 1 mL and analyzed by radio-HPLC on an Agilent 1260 Infinity system, using a gradient of 15-70% ACN (0.1% TFA) in 15 min at a flow rate of 2.0 mL/min.

***Therapeutic studies*** A431-CCK2R tumor bearing mice were randomized into vehicle control (n=6) and therapy (n = 8) cohorts. Treatment commenced when mice reached an average tumor volume of approximately 96 mm^3^ (range 63 – 133 mm^3^, 5-6 d after inoculation). Control mice were injected with 100 µL saline *via* the tail vein, while the therapy mice received [^177^Lu]Lu-DOTAGA-NPP (10 MBq x three consecutive days) or [^177^Lu]Lu-DOTA-MGS5 (30 MBq). Tumor size and body weight change were monitored until humane endpoint was reached. Tumor volume was determined by electronic caliper measurement using the formula V [mm^3^] = (length x width^2^)/2.

***Data and statistical analysis***. Unless otherwise stated, results are reported as mean ± standard deviation (SD). All statistical analyses were performed using GraphPad Prism (version 10.1.2, GraphPad Software, USA). Details about statistical tests are provided in the respective paragraphs.

## Results

### Synthesis and radiolabeling

All four nastorazepide-based ligands were obtained with ≥ 97% purity as determined by HPLC and characterized by ESI-MS. The chemical structures of all ligands investigated are shown in Figure 1. Analytical data are reported in Table 1.

***Radiolabeling.*** The radioligands, either labeled with ^111^In or with ^177^Lu, were obtained in high radiochemical purity (> 90%) and radiochemical yield (> 95%). The ^111^In-labeled ligands were used for the *in vitro* evaluation, and the ^177^Lu-labeled ligands were used in the *in vivo* evaluation.

Representative chromatograms, MS analyses and radio chromatograms of the extensively characterized ligands DOTAGA-NPP, DOTA-PP-F11 and DOTA-MGS5 are reported in Supplementary Information (Figures S1-S3).

### *In vitro* studies

Determination of the distribution coefficient. DOTAGA-conjugated ligands exhibited increased hydrophilicity relative to their DOTA analogues, while the NPS linker also yielded greater hydrophilicity compared to the NPP counterpart (Table 2). Overall, the hydrophilicity increased in the following order among the four radioligands: [^111^In]In-DOTA-NPP < [^111^In]In-DOTAGA-NPP < [^111^In]In-DOTA-NPS < [^111^In]In-DOTAGA-NPS. All four radioligands were more lipophilic compared to the reference [^111^In]In-DOTA-PP-F11 (log *D*_pH=7.4_ = -3.8±0.4).

***Cell association.*** All four ^111^In-labeled ligands demonstrated time-dependent and specific CCK2R-mediated cellular uptake. Among them, [^111^In]In-DOTAGA-NPP exhibited a cellular uptake (22.0 ± 0.2% of applied activity after 4 h at 37 °C) that was twice as high as that of the other three (Table 2) and significantly higher that of [^111^In]In-DOTA-PP-F11 (16.2 ± 1.8%, p = 0.047). The time dependent specific cellular uptake for each radioligand is reported in Figure S4. Specificity of the antagonist DOTAGA-NPP was confirmed by competitive blocking studies, in which cellular binding was reduced to < 2% of total added activity in the presence of CCK2R-specific competitors, namely Z-360, DOTA-MGS5, and gastrin-17 (Table S1).

***Determination of K_D_ and B_max_.*** The results of the saturation binding study (Table 2) indicated that the DOTAGA-conjugation improves affinity approximately 3-fold, compared to their DOTA-counterparts (K_D_ in the range of 7.8-9.6 nM vs. 23.8-30 nM, respectively). The affinity of both [^111^In]In-DOTAGA-derivatives was similar to [^111^In]In-DOTA-PP-F11 (K_D_ = 11.1 ± 2.5 nM). Importantly, all four antagonists had 3-4 times higher B_max_ compared to the agonist [^111^In]In-DOTA-PP-F11, independent of the chelator used (Table 2).

***Determination of antagonistic properties.*** IC_50_ values measured by the IP-One assay (Cisbio) indicated that the DOTA-conjugated ligands (DOTA-NPP: 0.08 ± 0.07 nM; DOTA-NPS: 0.12 ± 0.03 nM) have stronger antagonistic activity than their DOTAGA-counterparts (DOTAGA-NPP: 0.17 ± 0.10 nM; DOTAGA-NPS: 0.21 ± 0.07 nM). These results suggest that DOTA conjugation may enhance receptor antagonism in this assay system*.*

Serum protein binding. The protein bound fractions were < 8% for all the ^111^In-labeled ligands at any investigated time point (Table S2).

### *In vivo* studies

**Figure 2:**
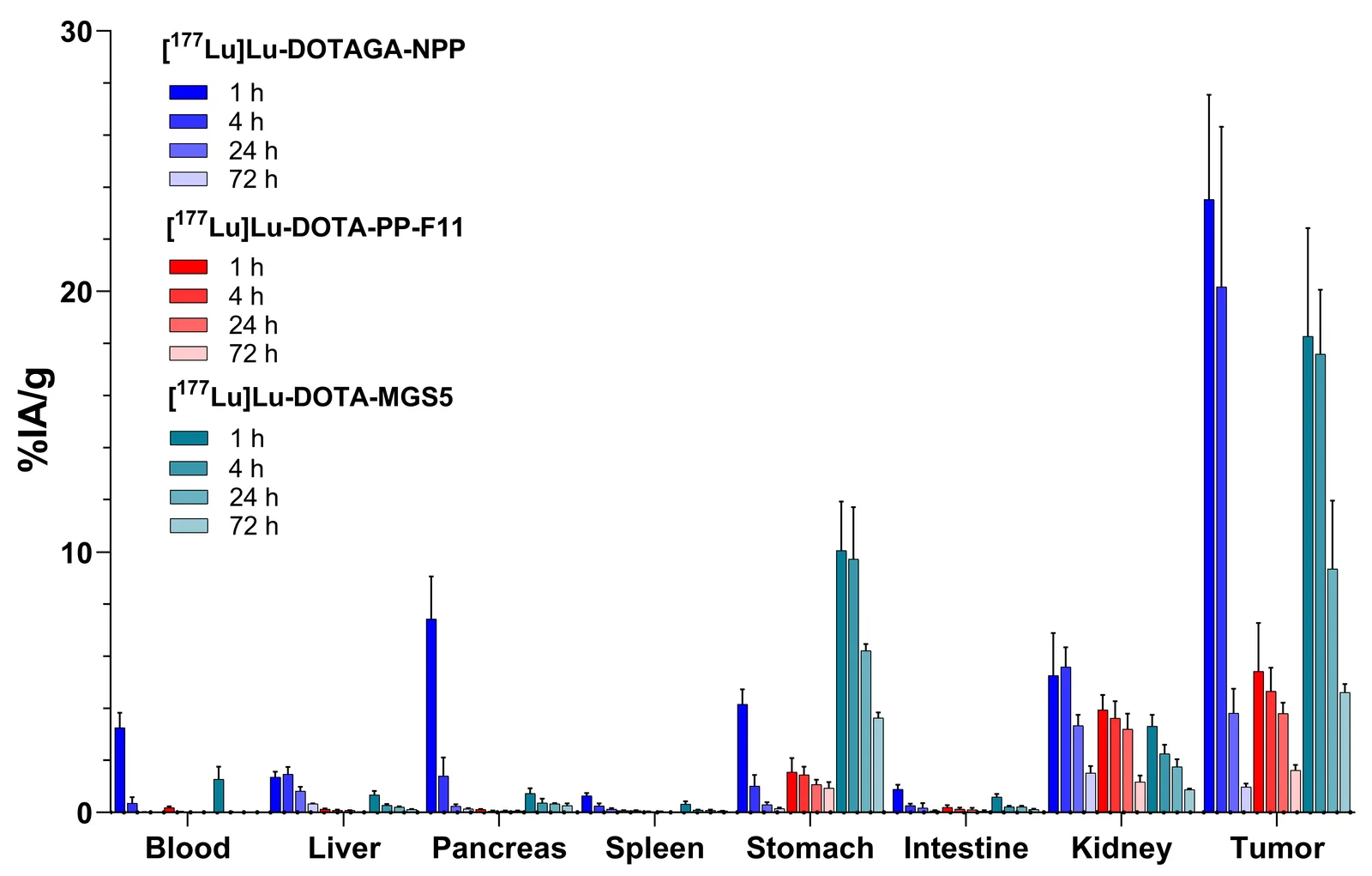
Biodistribution of [^177^Lu]Lu-DOTAGA-NPP, [^177^Lu]Lu-DOTA-PP-F11 and [^177^Lu]Lu-DOTA-MGS5 up to 72 h p.i. (20 pmol), (n = 3-8/group).

***Biodistribution studies*.** The comparative biodistribution results at 1 h post-injection (p.i.) showed high tumor uptake for all the tested Z360-based radioligands, ranging between 22.98 ± 7.04% IA/g and 29.00 ± 3.30% IA/g for [^177^Lu]Lu-DOTA-NPS and [^177^Lu]Lu-DOTA-NPP, respectively (Table 3). Notable uptake in blood-rich organs such as the heart, liver, and lungs, has been observed as well. In particular, the NPS derivatives exhibited substantially greater accumulation in the blood as well as in excretory organs such as the liver and kidneys. Studies at later time points revealed a progressive increase in liver uptake for [^177^Lu]Lu-DOTA-NPS (4.3 ± 1.0 and 7.2 ± 0.8 at 1 h and 4 h p.i., respectively), while the [^177^Lu]Lu-DOTAGA-NPS showed sustained kidney retention over 24 h p.i. (Table S3). The NPP derivatives were characterized by higher accumulation in the stomach and lower uptake in the excretory organs. Between the two, the DOTAGA derivative demonstrated the lower accumulation in blood and highly perfused organs and the best tumor-to-background ratios and was selected for further pharmacokinetic comparison against [^177^Lu]Lu-DOTA-PP-F11 and [^177^Lu]Lu-DOTA-MGS5 (Figure 2). Results for [^177^Lu]Lu-DOTAGA-NPP demonstrated pronounced tumor uptake at 1 h, a slight reduction at 4 h, and roughly 80% clearance within 24 h p.i. Head-to-head comparison with [^177^Lu]Lu-DOTA-MGS5 demonstrated no significant difference in tumor uptake values at 1 h p.i. (23.53 ± 4.02% IA/g *vs* 18.27 ± 4.16% IA/g, p = 0.18, for [^177^Lu]Lu-DOTAGA-NPP and [^177^Lu]Lu-DOTA-MGS5, respectively) while [^177^Lu]Lu-DOTA-PP-F11 exhibited significantly lower tumor uptake (5.42 ± 1.85% IA/g 1 h p.i.). The slower washout of [^177^Lu]Lu-DOTA-MGS5, resulted in a residual tumor activity of 4.61 ± 0.32% IA/g at 72 h p.i., compared to 0.99 ± 0.12% IA/g for [^177^Lu]Lu-DOTAGA-NPP. This slow washout of [¹⁷⁷Lu]Lu-DOTA-MGS5 was also found in the stomach, where uptake remained relatively high and persistent at all time points examined (10.07 ± 1.85% IA/g at 1 h p.i. and 1.97 ± 0.32% IA/g at 130 h p.i.), thereby negatively affecting its overall biodistribution profile. Full statistical comparison between [^177^Lu]Lu-DOTAGA-NPP and [^177^Lu]Lu-DOTA-MGS5 is reported in Figure S2. Results of the full biodistribution data for [^177^Lu]Lu-DOTAGA-NPP, [^177^Lu]Lu-DOTA-PP-F11 and [^177^Lu]Lu-DOTA-MGS5 are summarized in Supplementary Tables S4-S6.

**Figure 3:**
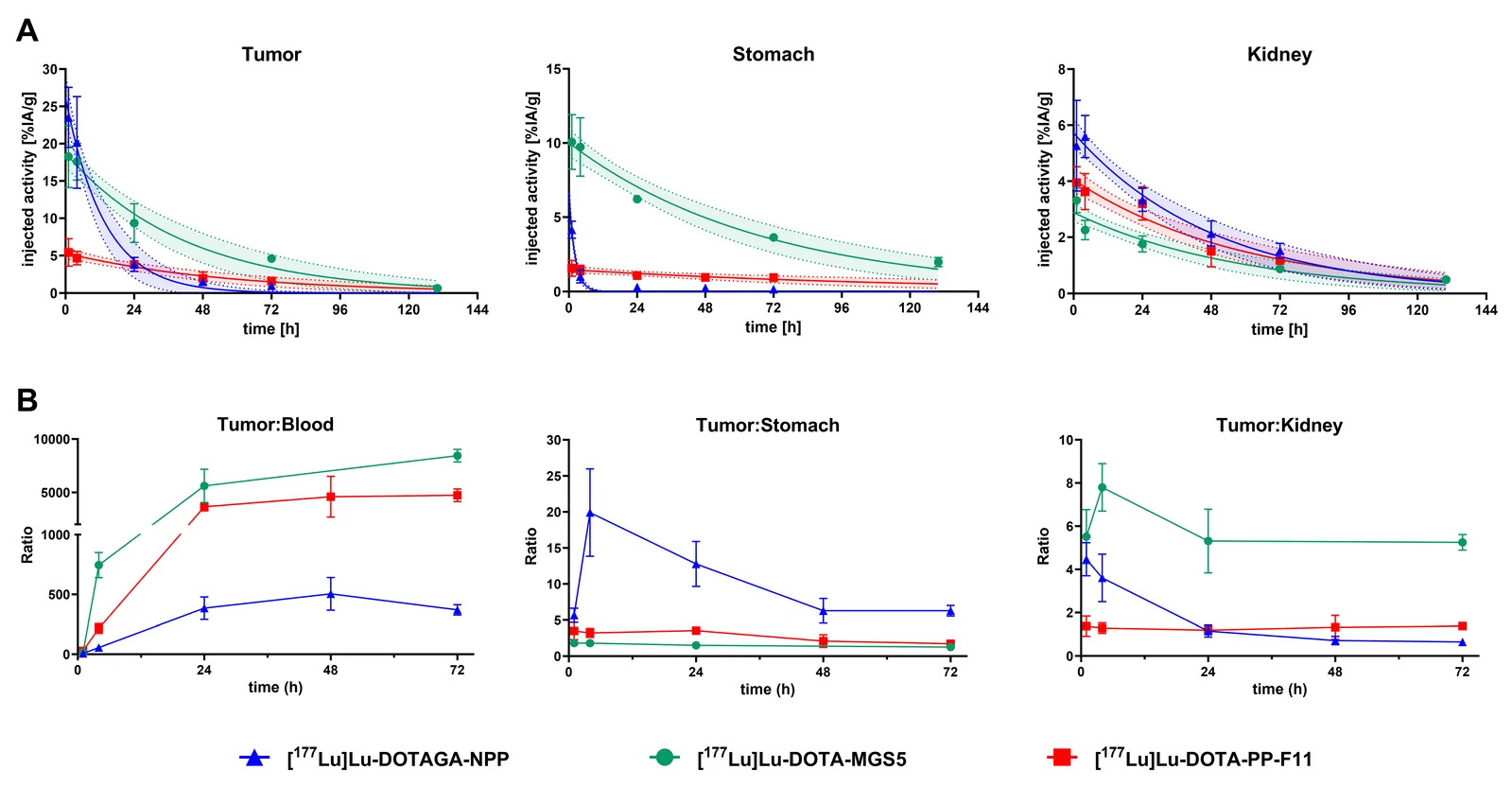
(A) Time-activity curves in tumor, stomach and kidney for [^177^Lu]Lu-DOTAGA-NPP, [^177^Lu]Lu-DOTA-MGS5 and [^177^Lu]Lu-DOTA-PP-F11. Single-exponential curve fits are shown with 95% confidence intervals. (B) tumor-to-organ ratios from 1 to 72 h p.i.

***Pharmacokinetics.*** Time-activity curves for tumor, stomach, and kidney were generated from biodistribution data using single-exponential fitting (Figure 3A). Tumor-to-organ ratios for the most relevant tissues are presented in Figure 3B. By combining the biological half-lives of the radioligands with the physical half-life of ^177^Lu, the area under the curve (AUC) was determined, and the resulting pharmacokinetic parameters are summarized in Table 4. The clearance of [^177^Lu]Lu-DOTAGA-NPP from the tumor proceeded with a half-life of 9.4 h (6.6 to 12.9 h; 95% CI), whereas [^177^Lu]Lu-DOTA-MGS5 and [^177^Lu]Lu-DOTA-PP-F11 exhibited longer tumor half-lives of 29.2 h and 39.4 h, respectively. In the kidneys, [^177^Lu]Lu-DOTAGA-NPP showed a half-life of 34.0 h, whereas the minigastrin analogues displayed slightly prolonged half-lives of 40.3 and 41.3 h for [^177^Lu]Lu-DOTA-MGS5 and [^177^Lu]Lu-DOTA-PP-F11, respectively. The most pronounced differences were observed in the stomach, where [^177^Lu]Lu-DOTAGA-NPP exhibited a rapid elimination half-life of 1.5 h, compared to substantially longer half-lives of 47.4 h and 84.8 h for [^177^Lu]Lu-DOTA-MGS5 and [^177^Lu]Lu-DOTA-PP-F11, respectively.

The different pharmacokinetic profiles were reflected in pronounced differences in AUC values. While the tumor AUC of [^177^Lu]Lu-DOTAGA-NPP was higher than [^177^Lu]Lu-DOTA-PP-F11 (333.2 ± 55.2 *vs* 243.5 ± 40.1% IA/organ x h), it was markedly lower than that of [^177^Lu]Lu-DOTA-MGS5 (667.5 ± 94.1% IA/g × h). In contrast, the stomach AUC of 14.0 ± 1.6% IA/g × h of the antagonist was markedly lower than that of both [^177^Lu]Lu-DOTA-MGS5 and [^177^Lu]Lu-DOTA-PP-F11 (529.0 ± 58.9 and 118.8 ± 24.6% IA/g × h, respectively). The kidney AUC of 231.5 ± 27.3% IA/g × h of [^177^Lu]Lu-DOTAGA-NPP was slightly higher than that of [^177^Lu]Lu-DOTA-MGS5 and [^177^Lu]Lu-DOTA-PP-F11 (134.0 ± 18.8 and 191.9 ± 24.6% IA/g × h, respectively), resulting in a tumor-to-kidney ratio that is in between [^177^Lu]Lu-DOTA-MGS5 and [^177^Lu]Lu-DOTA-PP-F11 (Figure 3B).

Specificity studies, reported in Table 5, demonstrated a more than 92% reduction in tumor uptake when a 1000-fold excess of DOTAGA-NPP or DOTA-MGS5 was pre-injected five minutes prior to the injection of [^177^Lu]Lu-DOTAGA-NPP (23.5 ± 4.0% IA/g vs. 1.7 ± 0.2% IA/g and 1.9 ± 0.4% IA/g, at 1 h p.i., respectively). Interestingly, pre-injection of the full ligand (DOTAGA-NPP) reduced tumor uptake by more than 97%, whereas the use of only the binding moiety (Z-360) resulted in a reduction of approximately 54% (20.2 ± 6.1% IA/g vs. 0.6 ± 0.1% IA/g and 9.2 ± 1.0% IA/g at 4 h p.i., respectively).

***Dosimetry***. Based on dosimetric extrapolation from mice to humans, the estimated absorbed doses were calculated and are presented in Table S7. An estimated radiation dose for the stomach wall in women/men were 0.018/0.014 mSv/MBq for [^177^Lu]Lu-DOTAGA-NPP, 0.181/0.138 mSv/MBq for [^177^Lu]Lu-DOTA-MGS5 and 0.002/0.002 mSv/MBq for [^177^Lu]Lu-DOTA-PP-F11. Corresponding kidney doses in women/ men were 0.301/0.238, 0.154/0.122 and 0.070/0.055 mSv/MBq, respectively.

**Figure 4:**
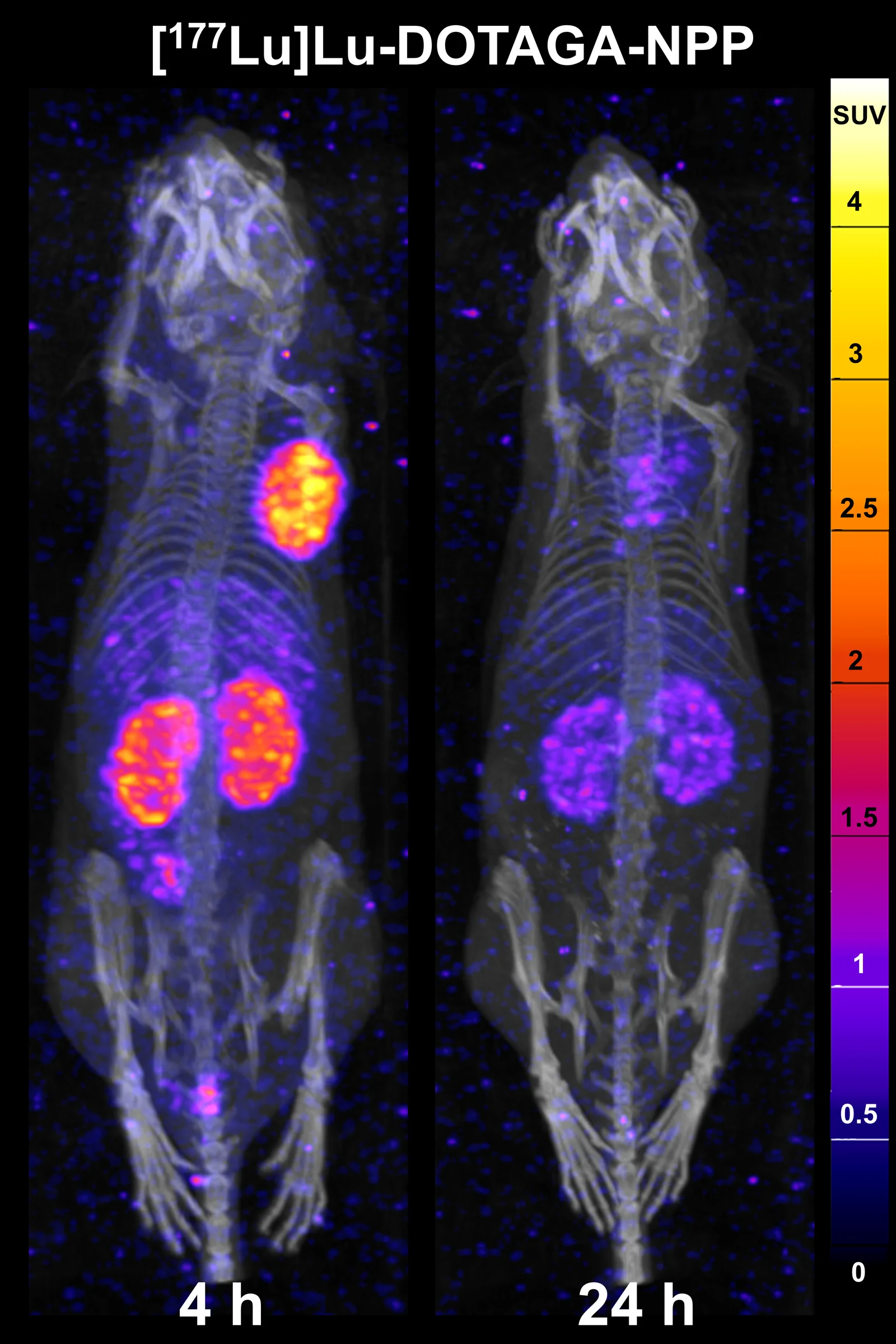
SPECT/CT images in A431-CCK2R tumor bearing mice, 4 h and 24 h p.i. of 50 pmol/10 MBq of [^177^Lu]Lu-DOTAGA-NPP.

***SPECT/CT imaging*.** SPECT/CT images of [^177^Lu]Lu-DOTAGA-NPP acquired at 4 h and 24 h p.i. (Figure 4) corroborated the biodistribution data, demonstrating high image contrast with distinct tumor delineation and noticeable uptake in kidneys and liver at 4 h p.i. Image contrast further increased at 24 h p.i. with comparable intensity in tumor and kidneys.

**Figure 5:**
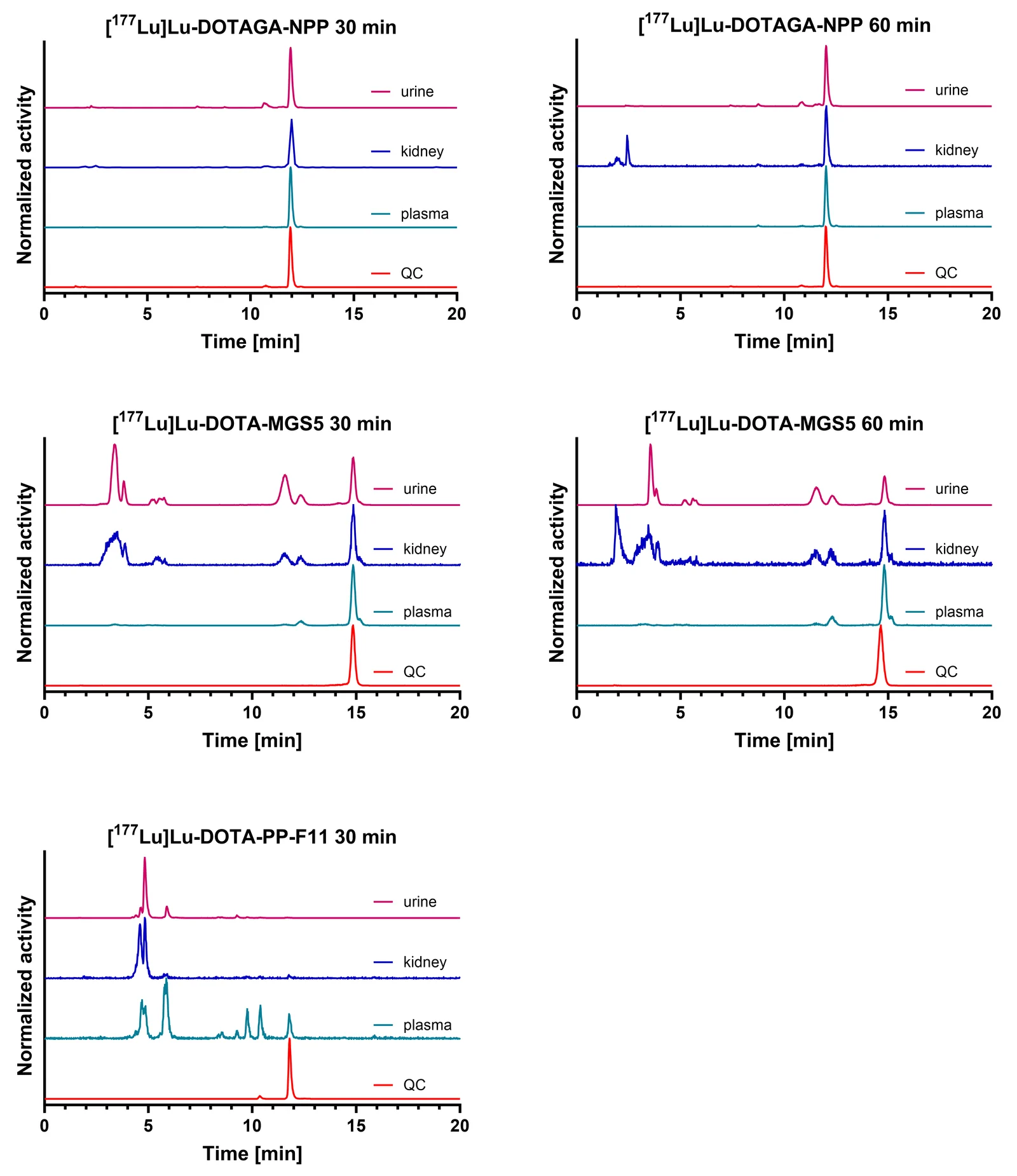
* In vivo* stability of the radioligands [^177^Lu]Lu-DOTAGA-NPP, [^177^Lu]Lu-DOTA-MGS5 and [^177^Lu]Lu-DOTA-PP-F11 in plasma, kidneys and urine (500 pmol, 25 MBq). Stability for [^177^Lu]Lu-DOTAGA-NPP, [^177^Lu]Lu-DOTA-MGS5 was determined 30 and 60 min after injection, due to the limited stability [^177^Lu]Lu-DOTA-PP-F11 was only investigated after 30 min p.i.

***In vivo metabolic stability*.** *In vivo* stability of the [^177^Lu]Lu-DOTAGA-NPP, [^177^Lu]Lu-DOTA-PP-F11 and [^177^Lu]Lu-DOTA-MGS5 was determined 30 min and 1 h after injection in plasma, kidneys and urine (Figure 5). At 1 h p.i., [^177^Lu]Lu-DOTAGA-NPP showed high stability in all the tissues (93.1%, 59.4%, and 84.1% intact radioligand in plasma, kidney and urine, respectively), while lower metabolic stability was found for [^177^Lu]Lu-DOTA-MGS5 (66.1%, 16.7%, and 20.2% intact radioligand in plasma, kidneys and urine, respectively). No intact [^177^Lu]Lu-DOTA-PP-F11 was detected in kidney homogenate and urine while < 10% was found in the plasma after 30 min p.i.

***Therapeutic efficacy***: Tumor growth inhibition was evaluated in A431-CCK2R⁺ xenografts with comparable baseline tumor volumes. To account for the distinctly different pharmacokinetic profiles, a fractionated dosing regimen of 3 × 10 MBq [^177^Lu]Lu-DOTAGA-NPP administered on consecutive days was compared with a single 30 MBq dose of [^177^Lu]Lu-DOTA-MGS5, alongside a control group receiving a single saline injection. Both radioligands were well tolerated. Although a slight and transient body weight loss was observed in both treatment groups, no animal exhibited a weight reduction exceeding 15% (Figure S3A).

A modest, statistically non-significant delay in tumor growth was observed in both treatment groups (Figure S3B). Consequently, median survival times of 8.5, 9, and 8.5 days for the saline, [^177^Lu]Lu-DOTAGA-NPP and [^177^Lu]Lu-DOTA-MGS5 groups, respectively, did not differ significantly (p = 0.50) (Figure S3C).

## Discussion

Clinical data on radiolabeled minigastrin analogs demonstrated promise for diagnosing CCK2R-expressing tumors such as medullary thyroid carcinoma (MTC), neuroendocrine tumors (NETs), and small cell lung cancer (SCLC). However, their therapeutic application is limited by low metabolic stability, high stomach radiation doses, and agonist-related side effects such as nausea and hypotension 27-29. These effects can be mitigated with CCK2R antagonists, and antagonist-based radioligands may overcome many of these limitations. Accordingly, in this study, we developed four new Z-360 based radioligands using either a peptidic (NPP) or a hydrophilic polysaccharide (NPS) linker 19, conjugated to DOTA or DOTAGA chelators. Although preclinical studies about CCK2R targeting antagonistic radioligand remain sparse, literature reports indicate that the choice of the linker and the chelator strongly affect affinity, hydrophilicity, and pharmacokinetics of Z-360 based radioligands 19, 21, 30, 31. Our goal is to create radioligands with optimal targeting for higher accumulation in the tumor, increased hydrophilicity for efficient urinary excretion, and versatility for labeling with various clinically relevant radiometals.

We observed clear hydrophilicity differences: the DOTAGA derivatives were more hydrophilic than the DOTA counterparts, likely due to the additional free carboxylic group which does not participate in the chelation of the radiometal and is ionized at physiological pH 32. Polysaccharide linkers further increased hydrophilicity. Furthermore, DOTAGA conjugation enhanced receptor affinity, achieving levels comparable to agonists and about three times higher than DOTA analogs, which translated into increased cellular uptake, particularly for [¹¹¹In]In-DOTAGA-NPP. All ligands behaved as antagonists and showed 2.5 to 4 times higher B_max_ than the agonist [^111^In]In-DOTA-PP-F11.

*In vivo*, these antagonists demonstrated excellent tumor uptake at 1 h p.i., although with elevated background activity. Their pharmacokinetics were linker-dependent: NPS-based ligands displayed increased blood-pool and renal activity, whereas NPP-based ligands showed more pronounced stomach and pancreas uptake. Since CCK2R is physiologically expressed in the stomach 33 uptake in this organs is expected and presents a key limitation of current minigastrin analogues 5, 6, 10. Among the tested ligands, [¹⁷⁷Lu]Lu-DOTAGA-NPP emerged as the most promising candidate, combining high tumor uptake with superior tumor-to-organ ratios (tumor-to-blood, tumor-to-liver, and tumor-to-kidney). The NPS-based ligands were excluded due to the persistent kidney uptake and unfavorable tumor-to-kidney ratios, while DOTAGA-NPP was preferred over DOTA-NPP because of its lower accumulation in blood and highly perfused organs, and improved tumor-to-organ ratios.

Blocking studies using a 1000-fold excess of DOTAGA-NPP, DOTA-MGS5 and Z-360 prior to administration of [^177^Lu]Lu-DOTAGA-NPP confirmed CCK2R-mediated tumor uptake. DOTAGA-NPP and DOTA-MGS5 reduced both tumor and stomach uptake to background levels, whereas Z-360 achieved only a partial blockade (46% and 35% reduction in tumor and stomach, respectively). In contrast, pancreatic uptake was reduced only by DOTAGA-NPP suggesting that pancreatic accumulation is not primarily CCK2 receptor-mediated and may involve interactions with the peptidic moiety of DOTAGA-NPP. Specificity was further confirmed *in vitro*, where the binding of [^177^Lu]Lu-DOTAGA-NPP to A431-CCK2R^+^ cells was efficiently blocked by Z-360, DOTA-MGS5 and gastrin-17.

The biodistribution profile of [^177^Lu]Lu-DOTAGA-NPP was benchmarked against the reference ligands [^177^Lu]Lu-DOTA-PP-F11 and [^177^Lu]Lu-DOTA-MGS5 under identical experimental conditions. Consistent with previous finding, ^177^Lu]Lu-DOTA-MGS5 showed superior tumor uptake compared with [^177^Lu]Lu-DOTA-PP-F11, but also its major limitation: high and persistent stomach accumulation. In contrast, [^177^Lu]Lu-DOTAGA-NPP effectively overcame this drawback by combining high tumor uptake with substantially reduced gastric accumulation and faster clearance, highlighting a key advantage of nastorazepide-based antagonists. Dosimetry extrapolation from biodistribution data indicated an approximately 10-fold lower absorbed stomach dose per unit administered radioactivity for [^177^Lu]Lu-DOTAGA-NPP compared with [^177^Lu]Lu-DOTA-MGS5. Although [^177^Lu]Lu-DOTAGA-NPP exhibited higher background activity, especially in highly perfused and excretory organs including the kidneys, the estimated absorbed dose remained well below toxicity thresholds, suggesting no expected dose-limiting renal toxicity under the studied conditions. While elevated background may reduce early imaging contrast, clear tumor visualization and positive tumor-to-kidney ratios were achieved at 4 and 24 h p.i.

Several factors may contribute to the observed pharmacokinetic profile, including lipophilicity, *in vivo* stability, plasma protein binding, perfusion, and metabolic processing. Although not of all parameters were systematically evaluated, *in vitro* study with ^111^In-labeled ligands showed consistently low plasma protein binding (< 8% bound fraction) for all ligands at all tested time points. *In vivo* metabolic stability further demonstrated superior stability of [^177^Lu]Lu-DOTAGA-NPP compared with [^177^Lu]Lu-DOTA-PP-F11 at 30 min p.i. consistent with previous findings by Kaloudi *et al.* 20. This represents a key advantage over minigastrin analogs, whose susceptibility to peptidase-mediated hydrolysis limits tumor uptake and retention 3. Comparison with the DOTA-MGS5 at 1 h p.i. also confirmed the higher *in vivo* stability of the antagonist, indicating that the rapid clearance of [^177^Lu]Lu-DOTAGA-NPP is unlikely due to insufficient metabolic stability. However, the mechanisms underlying its rapid tumor uptake and fast washout from tumors and CCK2R-positive organs remains to be elucidated.

This pharmacokinetic profile suggests that the ligand may not be optimally matched to the long physical half-life of ^177^Lu. Instead, it may be better suited for short-lived radionuclides, whose physical half-lives more closely match its rapid uptake and clearance kinetics. In the absence of preclinical therapy studies with CCK2R antagonists, we compared a fractionated regimen 3 × 10 MBq of the antagonist with a single administration of 30 MBq of [^177^Lu]Lu-DOTA-MGS5 to account for their different pharmacokinetics. Neither treatment produced a significant therapeutic effect versus saline controls, although both showed a small, non-significant growth inhibition. Although preclinical therapy studies of minigastrin analogues are limited, 37 MBq [^177^Lu]Lu-DOTA-CCK-66 significantly inhibited tumor growth in AR42J tumor xenografts 34, while [^177^Lu]Lu-DOTA-PP-F11 prolonged median survival in a therapeutic AR42J xenografts study 35. Interestingly, improved efficacy has also been observed for actinium-225 labeled minigastrin-analogues 34, 36. We therefore hypothesize that pairing DOTAGA-NPP with a therapeutic radionuclide featuring a physical half-life that better matches its fast pharmacokinetics could deliver a higher radiation dose to the tumor, while maintaining a favourable toxicity profile.

Comparison with previously reported nastorazepide-derived radioligands underscores the intricate influence of linker, chelator, and radiometal on pharmacokinetics, although differences in study design and animal models limit direct comparisons (Table S8). Our ligands share the linker used by Wayua *et al.* 19, but replace the N3S chelator with DOTA/DOTAGA. Similar to [^99m^Tc]Tc-CRL-3, polysaccharide-linker ligands showed low pancreas uptake (0.29 ± 0.19% IA/g and 0.56 ± 0.11% IA/g for [^99m^Tc]Tc-CRL-3 and [^177^Lu]Lu-DOTAGA-NPS, respectively) and distinct kidney accumulation (13.1 ± 2.0% IA/g and 18.1 ± 1.5% IA/g for [^99m^Tc]Tc-CRL-3 and [^177^Lu]Lu-DOTAGA-NPS, respectively). In contrast, the DOTA-conjugated Z-360 derivative [^111^In]In-IP-001 developed by Verona *et al.* 21 containing a hexanediamine-beta alanine-PEG_3_ linker, displayed high blood, intestinal, lungs, and pancreas activity with low specific tumor uptake. Using the same PEG_3_ linker coupled to Glu_4_-Lys, Kaloudi *et al.* 20 developed [^99m^Tc]Tc-DGA1 which achieved excellent tumor accumulation (31.6 ± 4.6% IA/g at 4 h p.i.) and reduced background but also high kidney retention (96.1 ± 12.0% IA/g at 4 h p.i.) and poor tumor-to-kidney ratio. Later, incorporation of a D-glutamic acid-PEG_3_ linker with DOTA, NODAGA and DOTAGA improved tumor to kidney ratio by almost 6-fold 31. Among the ^177^Lu-labeled DOTA and DOTAGA analogues (GAS1 and GAS3, respectively), GAS3 showed tumor uptake comparable to DOTAGA-NPP but with higher background activity, whereas GAS1 showed 2.4-fold lower tumor uptake; these differences were less pronounced ^111^In-labeled ligands. In contrast, our study found no major tumor uptake difference between ^177^Lu-labelled DOTA- and DOTAGA-derivatives, but DOTAGA-derivatives exhibited superior tumor-to-background ratios.

In conclusion, DOTAGA-NPP can be considered a lead Z-360-based radioligand, combining high tumor uptake, favorable pharmacokinetics, and improved tumor-to-stomach ratios. Although no significant antitumor effect was observed in the tested xenograft model, favorable tumor uptake and dosimetric estimates support further structural optimization of DOTAGA-NPP as a lead structure. Future efforts should focus on linker optimization to improve tumor retention, accelerate background clearance, and enhance overall therapeutic efficacy. Importantly, our study represents a significant step forward in the development of CCK2R-targeting radioligands and supports the feasibility of Z-360-based ligands as potential alternatives to minigastrin analogues.

## Supplementary Material

Supplementary material includes detailed synthetic procedures, analytical data, additional *in vitro* results, and biodistribution data.

## Figures and Tables

**Table 1:** Analytical data of studied ligands.

CODE	Ligand sequences	MW calculated	MW observed	Rt [min]
DOTA-NPS^a^	Z-360-8Aoc-DGlu-PS-DGlu-PS-DOTA	1933.14	1932 [M-H]^-^	19.5
DOTAGA-NPS^a^	Z-360-8Aoc-DGlu-PS-DGlu-PS-DOTAGA	2005.20	2004 [M-H]^-^	18.8
DOTA-NPP^b^	Z-360-Glu-Arg-DAsp-DOTA	1349.51	675.7 [M+2H]^2+^	14.8
DOTAGA-NPP^b^	Z-360-Glu-Arg-DAsp-DOTAGA	1421.58	711.7 [M+2H]^2+^	15.1
DOTA-PP-F11^b^	DOTA-(DGlu)_6_-Ala-Tyr-Gly-Trp-Met-Asp-Phe-NH_2_	2049.11	1025.4 [M+2H]^2+^	12.3
DOTA-MGS5^b^	DOTA-DGlu-Ala-Tyr-Gly-Trp-(N-Me)Nle-Asp-1Nal-NH_2_	1449.58	725.6 [M+2H]^2+^	14.3

^a^ Analytics performed following Method A; ^b^ Analytics performed following Method B (see Material and Methods in Supplementary Info). Rt: retention time; MW: molecular weight

**Table 2:** log *D*_pH=7.4_ values and affinity data for all ^111^In-labeled ligands.

Radioligand	log *D*_pH=7.4_ (mean ± SD)	% Cellular uptake (mean ± SD)	K_D_ [nM] (mean ± SD)	B_max_ [nM] (mean ± SD)
[^111^In]In-DOTA-NPP	-2.15 ± 0.01	11.0 ± 1.8	30.0 ± 1.77	1.19 ± 0.13
[^111^In]In-DOTAGA-NPP	-2.45 ± 0.14	22.0 ± 0.2	9.62 ± 3.76	1.08 ± 0.07
[^111^In]In-DOTA-NPS	-2.46 ± 0.03	10.4 ± 1.9	23.8 ± 1.94	0.72 ± 0.05
[^111^In]In-DOTAGA-NPS	-2.76 ± 0.04	11.4 ± 0.5	7.84 ± 3.95	0.86 ± 0.04
[^111^In]In-DOTA-PP-F11	-3.84 ± 0.43	16.2 ± 1.8	11.07 ± 2.52	0.29 ± 0.08

**Table 3:** Biodistribution data at 1h p.i. of 20 pmol (0.4-1.0 MBq) of the corresponding ^177^Lu-labeled ligands.

[^177^Lu]Lu	-DOTA-NPP	-DOTAGA-NPP	-DOTA-NPS	-DOTAGA-NPS
Organs	1 h p.i.			
Blood	5.86 ± 1.02	3.26 ± 0.57	9.03 ± 1.26	8.61 ± 0.96
Heart	2.08 ± 0.51	0.91 ± 0.21	2.98 ± 0.75	2.53 ± 0.19
Liver	3.32 ± 0.33	1.36 ± 0.21	4.29 ± 0.96	4.37 ± 0.69
Lung	4.31 ± 1.70	2.20 ± 0.23	5.48 ± 2.35	4.63 ± 0.53
Pancreas	13.11 ± 3.80	7.44 ± 1.63	2.14 ± 0.69	2.04 ± 0.16
Spleen	0.99 ± 0.22	0.65 ± 0.10	1.67 ± 0.50	1.74 ± 0.19
Stomach	5.73 ± 1.76	4.15 ± 0.57	2.38 ± 0.61	3.42 ± 0.34
Intestine	1.69 ± 0.15	0.90 ± 0.16	1.34 ± 0.07	2.19 ± 0.41
Adrenal	1.72 ± 0.50	1.05 ± 0.25	3.59 ± 1.61	3.50 ± 0.55
Kidney	6.55 ± 1.33	5.27 ± 1.62	8.87 ± 2.74	15.51 ± 1.67
Femur	1.00 ± 0.18	0.57 ± 0.10	1.84 ± 0.87	1.81 ± 0.14
Muscle	0.53 ± 0.08	0.41 ± 0.15	1.30 ± 0.39	1.34 ± 0.37
Tumor	29.00 ± 3.30	23.53 ± 4.02	22.98 ± 7.04	23.90 ± 3.81
Tumor to:	Ratios			
Blood	4.9	7.2	2.5	2.8
Liver	8.7	17.3	5.4	5.5
Kidney	4.4	4.5	2.6	2.6
Stomach	5.1	5.7	9.7	7.0

The results are expressed as %IA/g ± SD. (n=4, 4, 3, 5, 3, 3 mice/group, respectively).

**Table 4:** Pharmacokinetic parameters of [^177^Lu]Lu-DOTAGA-NPP, [^177^Lu]Lu-DOTA-MGS5 and [^177^Lu]Lu-DOTA-PP-F11.

		[^177^Lu]Lu-DOTAGA-NPP	[^177^Lu]Lu-DOTA-MGS5	[^177^Lu]Lu-DOTA-PP-F11
**Tumor**	R^2^	0.88	0.90	0.72
	t_1/2_ [h] (range)	9.3 (6.6 - 12.9)	29.2 (20.4 - 40.9)	39.4 (27.2 - 61.0)
	AUC [%IA/g × h] ± SE	333.2 ± 55.2	667.5 ± 94.1	243.5 ± 40.1
				
**Kidney**	R^2^	0.79	0.85	0.81
	t_1/2_ [h] (range)	34.0 (26.1 – 45.5)	40.3 (27.3 – 58.4)	41.3 (31.1 – 57.2)
	AUC [%IA/g × h] ± SE	231.5 ± 27.3	134.0 ± 18.8	191.9 ± 24.6
				
**Stomach**	R^2^	0.94	0.88	0.41
	t_1/2_ [h] (range)	1.5 (1.2 - 1.8)	47.4 (35.2 – 63.9)	84.8 (49.9 – 215.8)
	AUC [%IA/g × h] ± SE	14.0 ± 1.6	529.0 ± 58.9	118.8 ± 24.6

**Table 5:** Blocking studies of [^177^Lu]Lu-DOTAGA-NPP with pre-injection of 1000-fold excess of competitor.

	DOTAGA-NPP		DOTA-MGS5	Z-360
Organs	1 h p.i.	4 h p.i.	1 h p.i.	4 h p.i.
Blood	3.21 ± 0.41	0.13 ± 0.01	3.17 ± 0.41	0.35 ± 0.08
Heart	0.82 ± 0.15	0.10 ± 0.03	0.87 ± 0.16	0.13 ± 0.03
Liver	1.60 ± 0.28	1.39 ± 0.33	1.54 ± 0.29	1.34 ± 0.10
Lung	2.04 ± 0.21	0.42 ± 0.00	2.10 ± 0.44	0.56 ± 0.35
Pancreas	3.73 ± 0.34	0.64 ± 0.21	8.70 ± 1.43	1.51 ± 0.26
Spleen	0.63 ± 0.07	0.20 ± 0.08	0.61 ± 0.09	0.20 ± 0.02
Stomach	1.04 ± 0.13	0.49 ± 0.24	0.98 ± 0.07	0.35 ± 0.08
Intestine	0.93 ± 0.14	0.53 ± 0.47	0.86 ± 0.17	0.23 ± 0.01
Adrenal	1.25 ± 0.31	0.35 ± 0.06	0.86 ± 0.11	0.38 ± 0.05
Kidney	6.30 ± 1.00	4.25 ± 0.15	6.62 ± 0.82	3.83 ± 0.67
Femur	0.49 ± 0.11	0.11 ± 0.02	0.57 ± 0.06	0.12 ± 0.01
Muscle	0.37 ± 0.05	0.06 ± 0.01	0.39 ± 0.01	0.07 ± 0.03
Tumor	1.70 ± 0.22	0.61 ± 0.22	1.88 ± 0.40	9.20 ± 0.97

The results are expressed as %IA/g ± SD. n=3 mice/group.

## Data Availability

Data are presented in the main text or supplementary information. Additional data are available fromthe corresponding author upon request.

## Abbreviations

ACN: acetonitrile
B_max_: maximum binding capacity
CCK2R: cholecystokinin-2 receptor
CCK: cholecystokinin
MTC: medullary thyroid carcinoma
SCLC: small cell lung cancer
MG: minigastrin
DTPA: diethylenetriaminepentaacetic acid
CT: computed tomography
DOTA: 1,4,7,10-tetraazacyclododecane-1,4,7,10-tetraacetic acid
Fmoc: 9-fluorenylmethoxycarbonyl
DOTAGA: (1,4,7,10-tetrakis(carboxymethyl)-1,4,7,10-tetraazacyclododecane glutaric acid)
ESI-MS: electrospray ionization mass spectrometry
GPCR: G protein-coupled receptors
K_D_: dissociation constant
MIP: maximum intensity projection
PBS: phosphate-buffered saline
p.i.: post injection
PEG: poly(ethylene glycol)
RT: room temperature
PET: positron emission tomography
RCP: radiochemical purity
RP-HPLC: reversed phase high performance liquid chromatography
SPECT: single photon emission tomography
TFA: trifluoroacetic acid
