# Supplementary material for: Novel nastorazepide-based radioligands demonstrate effective cholecystokinin-2 receptor targeting with potential for theranostic applications
Source: Theranostics. 2026 Jul 29;16(15):8447–60. doi: 10.7150/thno.129327 (PMC13549144; doi:10.7150/thno.129327)
Supplement: Supplementary file 1 [file thnov16p8447s1.pdf]

Supplementary information

Novel nastorazepide-based radioligands demonstrate effective cholecystokinin-2 receptor targeting with potential for theranostic applications

Lennart Bohrmann[1#], Doroteja Novak[2,3#], Marko Kroselj[2,3], Raghuvir H. Gaonkar[1], Francesca Porto[1], Melpomeni Fani[1], Petra Kolenc[2,3], Marko Anderluh[3*], Rosalba Mansi[1*]

[1]Division of Radiopharmaceutical Chemistry, University Hospital Basel, Petersgraben 4, 4031 Basel, Switzerland

[2]Division of Nuclear Medicine, University Medical Centre Ljubljana, Zaloška 7, 1000 Ljubljana, Slovenia

[3]Department of Pharmaceutical Chemistry, Faculty of Pharmacy, University of Ljubljana, Aškerčeva 7, 1000 Ljubljana, Slovenia

[#]These authors contributed equally to this work

Corresponding authors:

*Mansi Rosalba: rosalba.mansi@usb.ch; +4161 3285628

*Anderluh Marko: marko.anderluh@ffa.uni-lj.si, +386 1 476 9639

Materials and methods

General information. All commercial chemicals (analytical grade) were used without further purification if not otherwise stated. [^{111}In]InCl$_3$ was obtained from Mallinckrodt Medical (Petten, The Netherlands). [^{177}Lu]LuCl$_3$ (non-carrier added) was kindly provided by ITM (Munich, Germany). Fmoc-protected solid-phase synthesis building blocks were purchased from Iris Biotech GmbH (Marktredwitz, Germany), Z-360 was purchased from MedKoo Biosciences (Chapel Hill, North Carolina, USA), DO3AtBu-N-(2-aminoethyl)ethanamide and DOTAGA(tBu)$_4$-NH$_2$ were from Macrocyclics (Texas, USA) or Chematech (Dijon, France). All other reagents and solvents were purchased from Sigma-Aldrich (St. Louis, Missouri, United States), Alkaloid (Skopje, North Macedonia) and Merck (Darmstadt, Germany). Analyses were performed by high-performance liquid chromatography (HPLC) using either an Agilent 1110 system (Agilent Technologies, USA) equipped with a Kinetex C18 column (5 µm, 110 Å, 250 × 4.6 mm; Phenomenex, USA) or an LC-MS-2020 system (Shimadzu, Japan) equipped with a Jupiter Proteo C12 column (90 Å, 4 µm, 250 × 4.6 mm; Phenomenex, USA). For the Agilent system, gradient elution was carried out from 10–80% acetonitrile (ACN) with 0.1% trifluoroacetic acid (TFA) over 25 min at a flow rate of 0.75 mL/min, followed by electrospray ionization mass spectrometry (ESI-MS) analysis using an Exactive™ Plus Orbitrap Mass Spectrometer (Thermo Scientific™, Thermo Fisher Scientific, USA) (Method A). For the Shimadzu LC-MS system, the gradient was 20–80% ACN with 0.1% TFA over 15 min at a flow rate of 1 mL/min (Method B). Purification was performed by semi-preparative RP-HPLC using a Phenomenex Kinetex XB-C18 column (5 µm, 110 Å, 250 × 10 mm). Elution was carried out with a linear gradient of 20–60% or 20–80% ACN containing 0.1% TFA over 25 min at a flow rate of 3.5 mL/min.

The radiochemical purity of the ^{111}In-labeled ligands was evaluated on an Agilent 1110 HPLC system (Agilent Technologies) equipped with an in-line NaI radiodetector (Raytest, Germany). Analyses were performed on either a Kinetex C18 column (5 µm, 110 Å, 250 × 4.6 mm) or a Gemini C18 column (5 µm, 110 Å, 150 × 4.6 mm). In both cases, a 10–60% gradient of ACN containing 0.1% TFA was applied over 25 min at a flow rate of 0.75 mL/min.

For the quality control of the ^{177}Lu-labeled ligands, radio-HPLC were conducted on a HPLC-Agilent 1260 Infinity system having a Berthold LB509 γ-detector and using a Proteo Jupiter C-12 column (4 x 250 mm, 4 µm particle size). The gradient elution of 15–70% ACN (0.1% TFA)

in 15 min was applied with a flow rate of 2.0 mL/min. Quantitative γ-counting was performed using a Hidex Automatic gamma counter (Hidex, Finland) and a COBRA 5003 well-type γ-counter (Packard Instruments,USA).

Synthesis. The peptides were synthetized by acid-sensitive 2-chlorotryl chloride resin (2-CTC). The first Fmoc-protected amino acid was coupled using 4 equivalents of DIPEA. Unreacted functionalities were capped with a DCM/MeOH/DIPEA solution (17/2/1), followed by Fmoc deprotection. Resin loading was determined then estimated spectrophotometrically by quantifying the released Fmoc group at 300 nm. For the NPP-based peptides, the first amino acid was Fmoc-D-Asp(OtBu)-OH, followed by coupling with Fmoc-Arg(Pbf)-OH, Fmoc-Glu(OtBu)-OH and Z-360 (MedKoo, USA) in presence of HATU (3 equiv.) and DIPEA (5 equiv.). For the NPS-based peptides, the first amino acid Fmoc-3,4,5,6-di-O-isopropylidene-1-amino-1-deoxy-D-glucitol-γ-glutamate was load on the resin, followed by Fmoc-Glu(OtBu)-OH, Fmoc-3,4,5,6-di-O-isopropylidene-1-amino-1-deoxy-D-glucitol-γ-glutamate, Fmoc-Glu(OtBu)-OH, Fmoc-8-Aoc-OH (Fmoc-8-amino-octanoic acid) and Z-360, as reported previously [1]. The peptides were cleaved fully protected using a solution of 1% TFA in DCM after precipitation in ice-cold water. DOTA or DOTAGA (1.2 equiv.) was coupled in solution, in dry DMF under an argon atmosphere, in presence of HATU (2.4 eq) and DIPEA (3.6 eq). Upon completion of the reaction, the product was isolated by extraction followed by solvent removal. Side-chain protecting groups were then removed using a TFA/tioanisole/triisopropylsilane/H_2O mixture (92.5/2.5/2.5/2.5).The deprotected product was precipitated with cold diethyl ether, purified and characterized *via* RP-HPLC and ESI-MS.

Serum protein binding. Serum protein binding was determined using fresh human serum. 1 mL of human serum was pre-adjusted at 37 °C, 5% CO_2 for 1 h. Radiolabelled compound (10 µL, approx. 0.3 MBq/µL, 0.04 µg/µL) was added to serum, vigorously shaken and incubated 37 °C, 5 % CO_2 for 0, 1, 4 and 24h. At each time point, 33 µL of full serum were withdrawn and the radioactivity was counted in gamma counter. To determine the protein bound fraction, 100 µL of serum were treated with 200 µL MeOH to precipitate the proteins, then the sample was centrifuged and 100 µL of supernatant was withdrawn to count the radioactivity in gamma counter. The percentage of serum protein bound fraction was calculated as the ratio between the bound radioactivity and total radioactivity (in full serum). The experiment was carried out in duplicate.

Determination of antagonistic activity. Antagonistic activity of the ligands was evaluated by determining IC_{50} values using Cisbio IPOne® assay (Cisbio Perkin Elmer, USA) on A431-CCK2R cells. Gastrin-17 (G17) was used as the agonist at its previously determined EC_{90}. [2]. The suspension protocol for biologically adherent cells from the producer's guide for optimization was used. Cells were detached with 0.25% trypsin, washed with PBS (pH 7.4), and resuspended in assay buffer. Approximately 5000 cells/well were incubated with test ligands (final concentrations ranging from 10^{-6} to 10^{-12} M) for 1 h at 37 °C, followed by the addition of G17 and a further 1 h incubation. Lysis and detection reagents containing labeled IP-1 and anti-IP-1 antibodies were then added, and time-resolved fluorescence was measured at 665 nm and 620 nm after 1 h using a Tecan Spark reader. Fluorescence ratios were converted to IP-1 concentrations using a standard calibration curve, to generate concentration-response curve. Untreated cells served as negative control, while Z-360 in presence of G17 was used as positive control. IC_{50} values were calculated in GraphPad Prism 6.00 (GraphPad Software, USA). The experiments were performed in triplicates.

SPECT/CT imaging study. SPECT/CT images were acquired for [^{177}Lu]Lu-DOTAGA-NPP (100 μL, 200 pmol/5–6 MBq) at 4 h and 24 h p.i. Mice were imaged in a supine, head-first position using a dedicated small-animal SPECT/CT system (NanoSPECT/CT™, Mediso, Budapest, Hungary). Whole-body CT was acquired first (45 kVp, 177 μA; 90 s; 180 projections/rotation; pitch 1), followed by a helical SPECT scan from head to toe using APT1 multi-pinhole collimators. SPECT data were collected over 24 projections (200 s each), ensuring ≥50 kilocounts per projection. Reconstruction was performed using HiSPECT software (v1.4.1876) with the manufacturer's iterative algorithm (3 subsets, 9 iterations, 35% filtering; 128 × 128 matrix; 0.3 mm pixels). CT images were reconstructed using CTReco (r1.146) with filtered back projection (Ram-Lak filter, 100% cut-off; 0.2 mm pixel size). Fused datasets were analyzed in orthogonal views and maximum intensity projections using InVivoScope (v1.43, Mediso).

Results

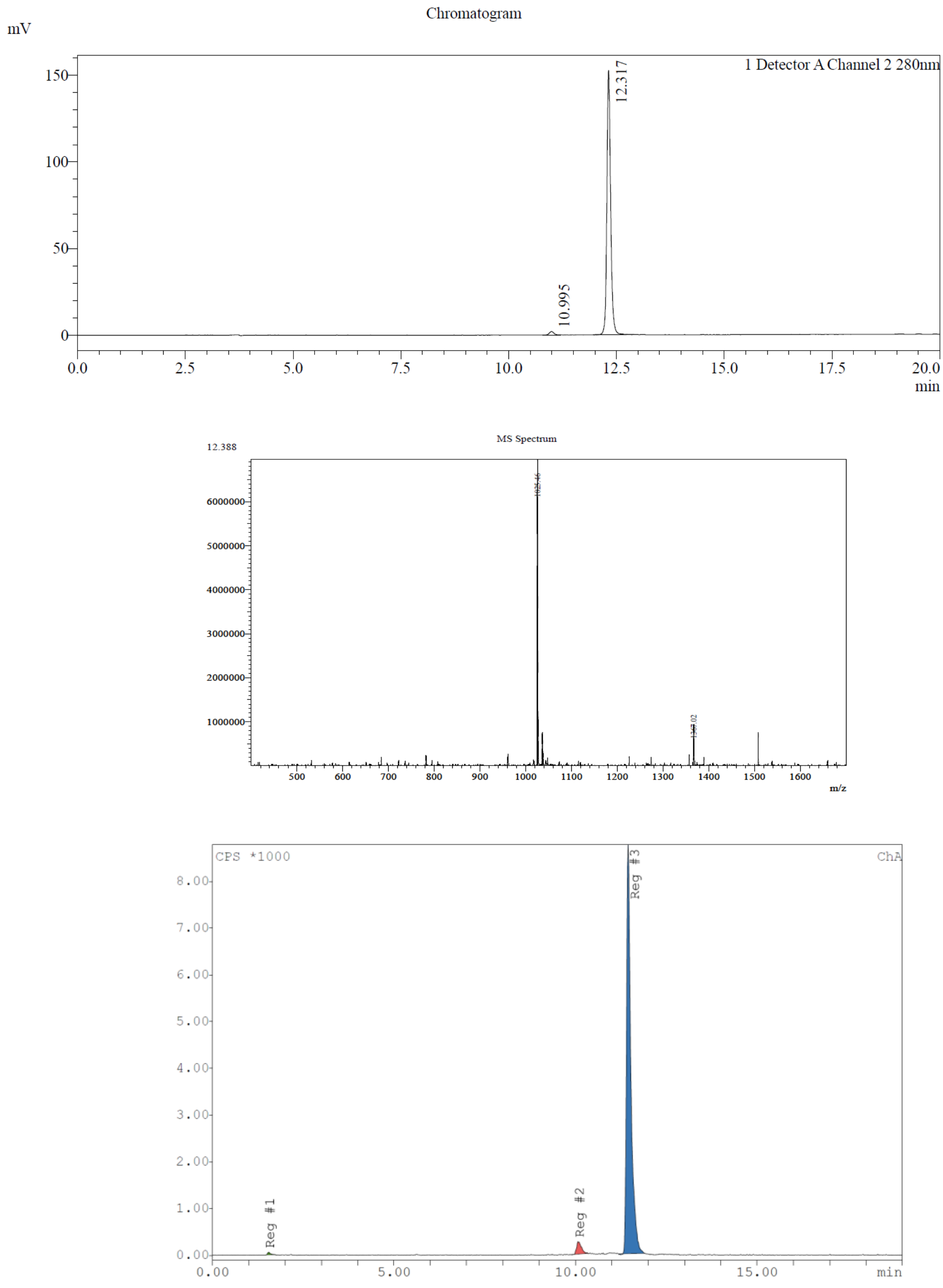

Figure S1. Representative examples of RP-HPLC chromatograms of DOTA-PP-F11, corresponding MS spectrum and radio-chromatogram of [^{177}Lu]Lu-DOTA-PPF11 (RCP:96.4%)

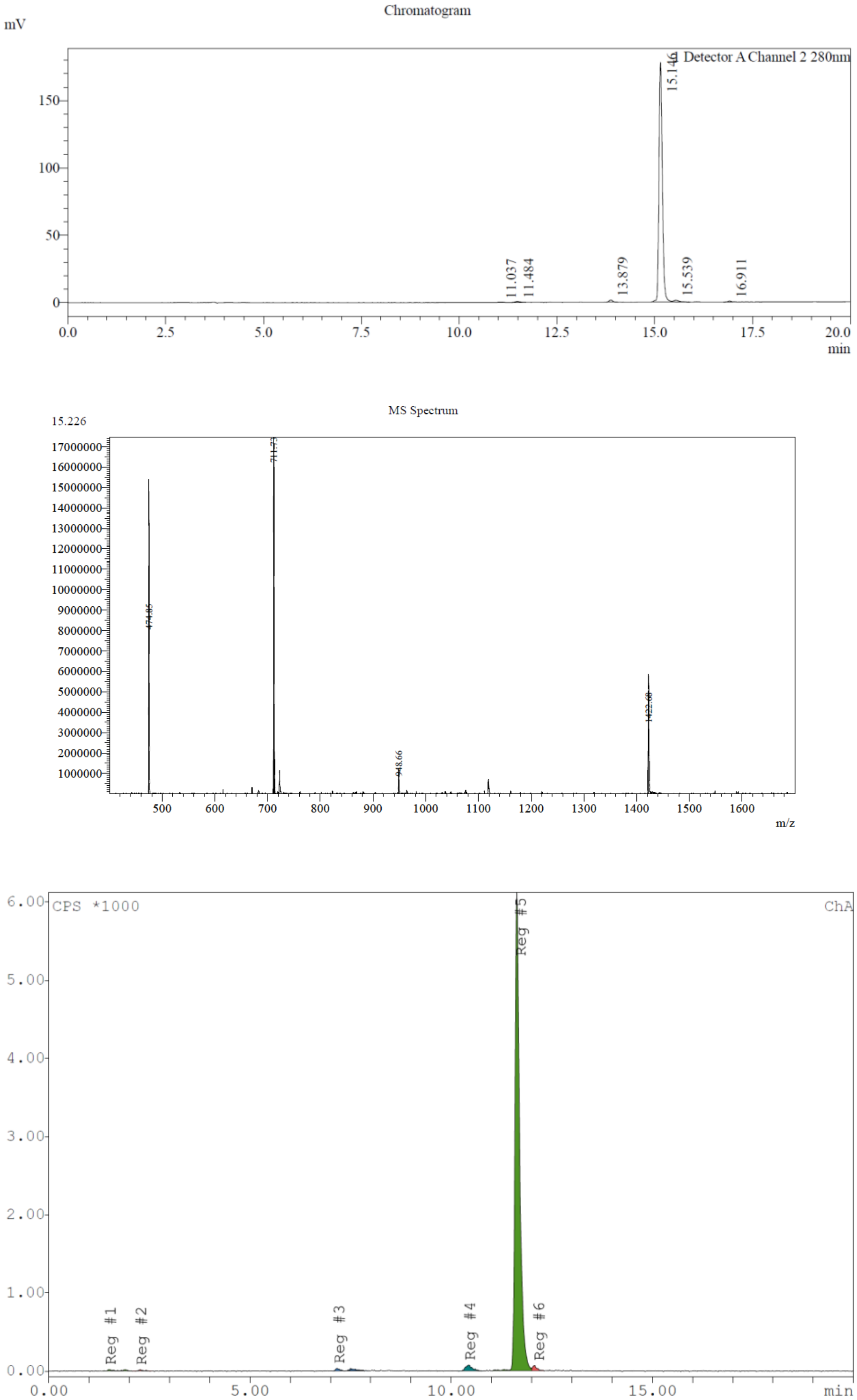

Figure S2. Representative examples of RP-HPLC chromatograms of DOTAGA-NPP, corresponding MS spectrum and radio-chromatogram of [^{177}Lu]Lu-DOTAGA-NPP (RCP:95.8%).

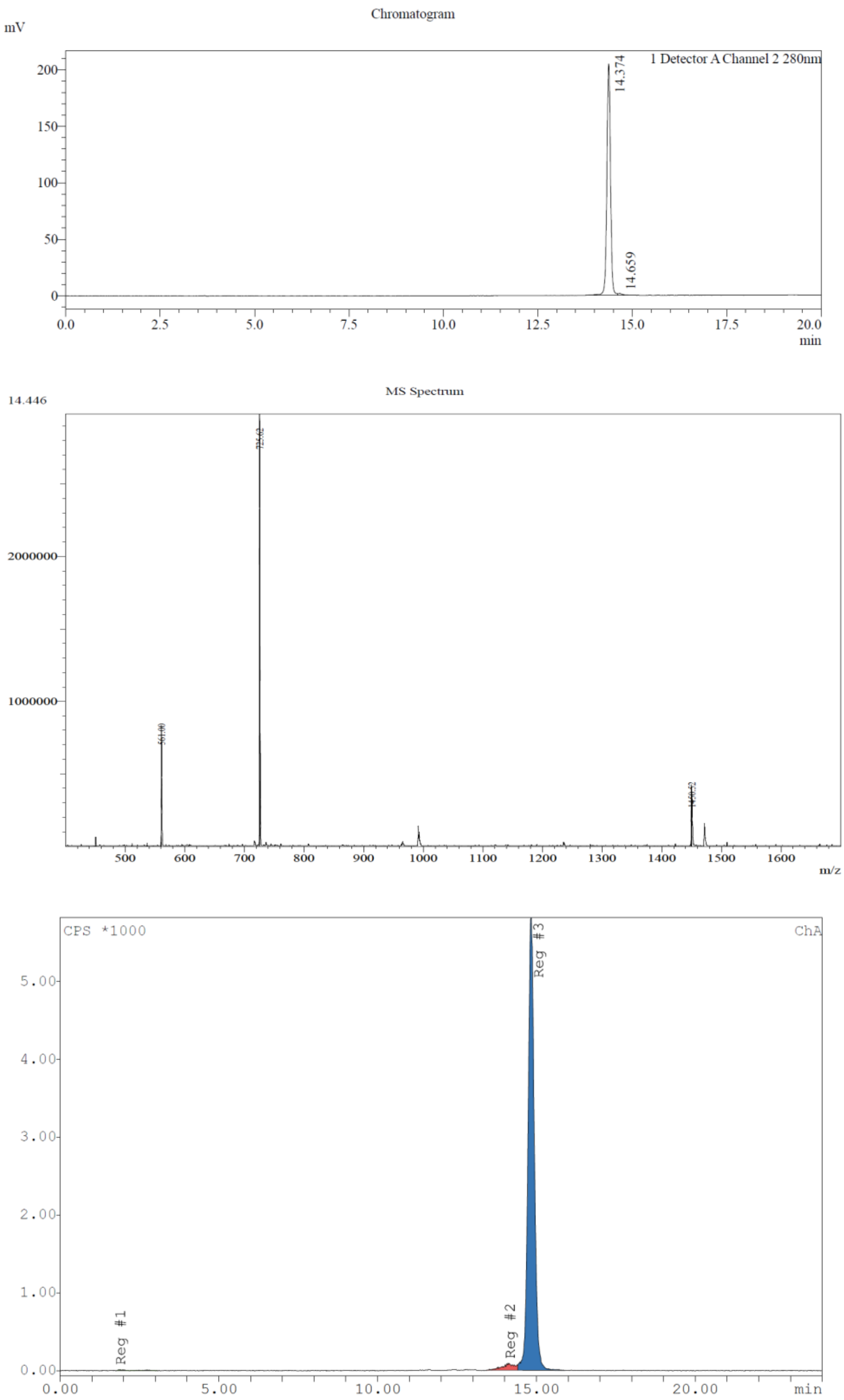

Figure S3. Representative examples of RP-HPLC chromatograms of DOTA-MGS5, corresponding MS spectrum and radio-chromatogram of [^{177}Lu]Lu-DOTA-MGS5 (RCP:96.8%).

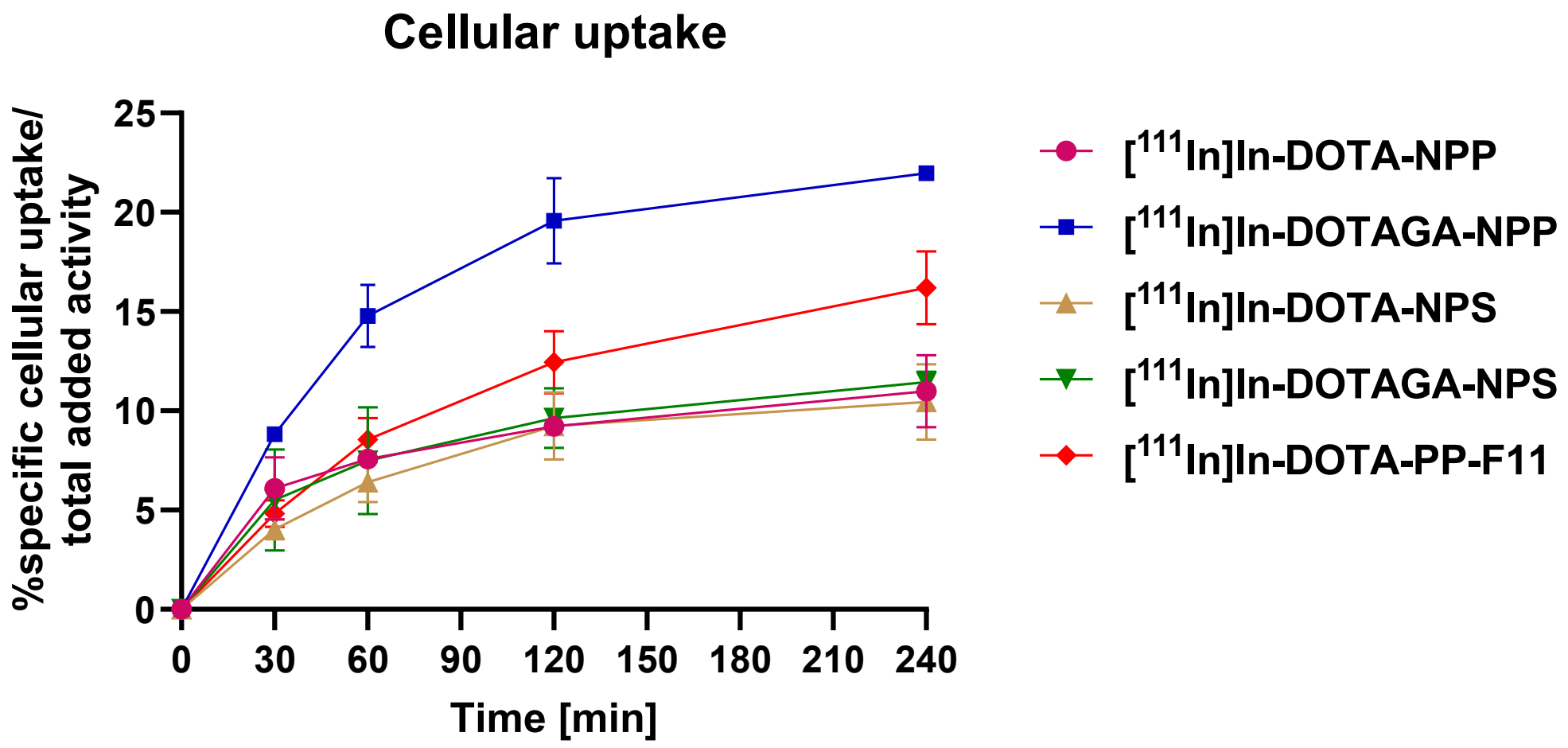

Figure S4. Specific total cellular uptake of the radioligands observed after incubation on A431-CCK2R cells at 37°C for different time points (30, 60, 120 and 240 min).

Table S1. Cellular uptake rate of [^{177}Lu]Lu-DOTAGA-NPP blocked with: Z360, DATA-MGS5 and Gastrin-17

[^{177}Lu]Lu-DOTAGA-NPP		
**Blocking agent**	**Total Cellular uptake %**	**Non-Spec Cellular uptake %**
Z-360	35.3 ± 1.8	0.8 ± 0.3
DOTA-MGS5	35.7 ± 1.5	0.8 ± 0.03
Gastrin-17	38.6 ± 1.8	1.9 ± 0.08

Table S2. Protein bound fraction expressed as % of the total added activity

**Time (h)**	**[^{111}In]In-DOTA-PP-F11**	**[^{111}In]In-DOTA-NPP**	**[^{111}In]In-DOTAGA-NPP**	**[^{111}In]In-DOTA-NPS**	**[^{111}In]In-DOTAGA-NPS**
0	5.6 ± 1.7	3.7 ± 1.0	4.9 ± 0.5	4.9 ± 1.6	2.2 ± 1.3
1	10.4 ± 1.6	3.5 ± 0.7	4.0 ± 2.6	2.0 ± 2.2	1.5 ± 1.2
4	7.0 ± 1.1	3.1 ± 1.2	3.4 ± 0.8	1.4 ± 0.6	0.8 ± 0.1
24	9.1 ± 1.6	5.3 ± 1.7	3.8 ± 0.2	1.7 ± 1.5	2.8 ± 0.9

Table S3. Biodistribution data at 4 h and 24 h p.i. of 20 pmol (0.4-1.0 MBq) of [177Lu]Lu-DOTA/DOTAGA-NPS.

	**[177Lu]Lu-DOTAGA-NPS**		**[177Lu]Lu-DOTA-NPS**
Organs	4 h p.i.	24 h p.i.	4 h p.i.
Blood	1.89 ± 0.28	0.02 ± 0.003	5.16 ± 0.41
Heart	0.66 ± 0.11	0.13 ± 0.02	1.64 ± 0.28
Liver	2.40 ± 0.34	1.61 ± 0.17	7.20 ± 0.78
Lung	1.26 ± 0.22	0.16 ± 0.02	2.94 ± 0.17
Pancreas	0.56 ± 0.11	0.11 ± 0.02	1.35 ± 0.10
Spleen	0.85 ± 0.25	0.33 ± 0.03	2.35 ± 0.43
Stomach	1.11 ± 0.25	0.34 ± 0.03	1.99 ± 0.31
Intestine	0.54 ± 0.16	0.18 ± 0.04	1.13 ± 0.07
Adrenal	1.58 ± 0.50	0.69 ± 0.12	2.43 ± 0.53
Kidney	18.09 ± 1.51	15.12 ± 2.65	14.91 ± 0.36
Femur	0.67 ± 0.17	0.24 ± 0.01	1.28 ± 0.26
Muscle	0.31 ± 0.09	0.067 ± 0.01	0.69 ± 0.09
Tumor	**18.33 ± 4.36**	**4.89 ± 0.66**	**24.23 ± 1.97**

The results are expressed as %IA/g±SD (n=5, 3, 3 mice/group, respectively).

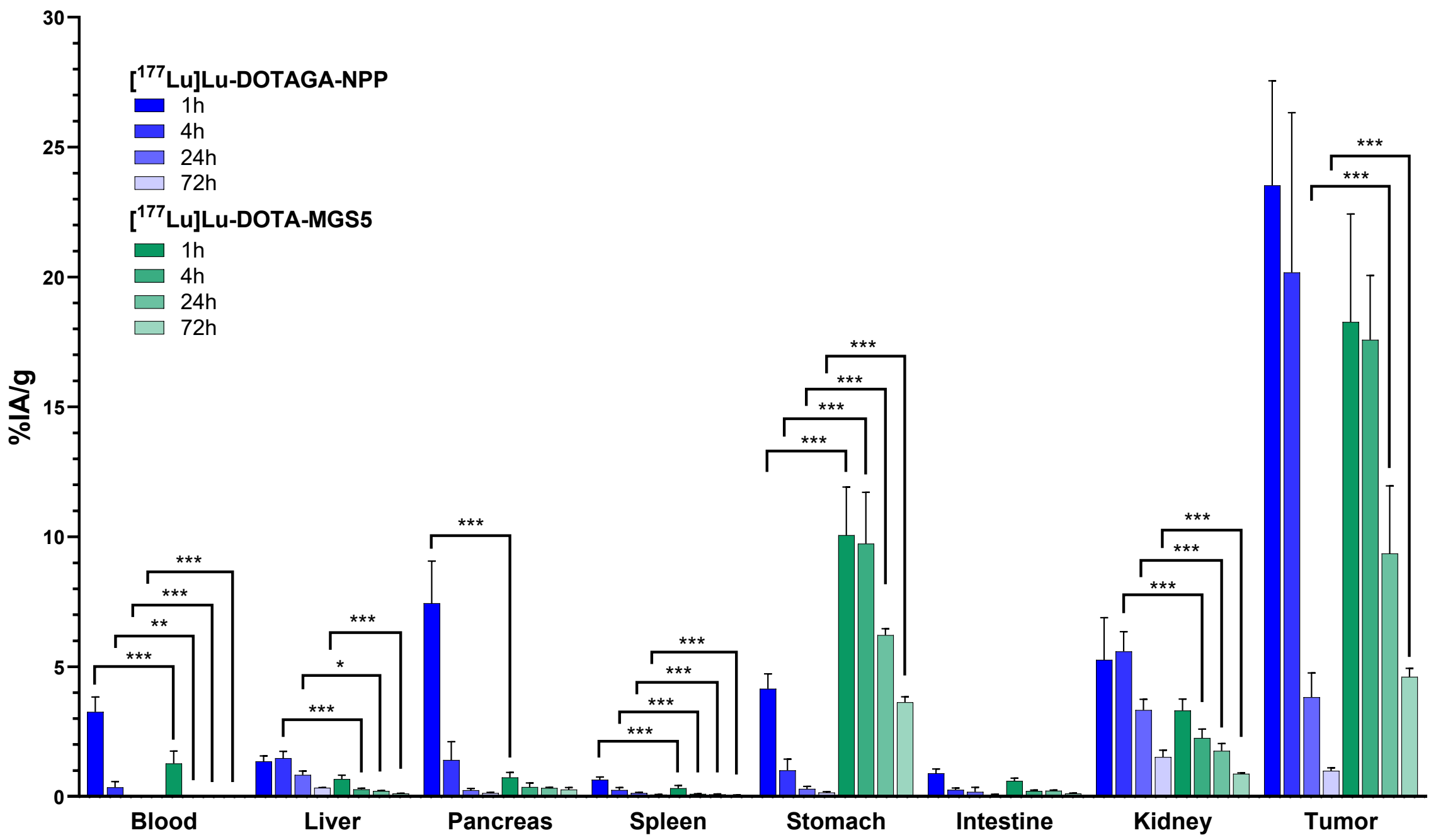

Figure S5. Comparison of [177Lu]Lu-DOTAGA-NPP and [177Lu]Lu-DOTA-MGS5. Data are represented as mean ± SD. Only statistically significant findings are reported (* = p ≤ 0.05; ** = p ≤ 0.01; *** = p ≤ 0.001).

Table S4. Biodistribution data of 20 pmol (0.4-1.0 MBq) of [^{177}Lu]Lu-DOTAGA-NPP.

	**[^{177}Lu]Lu-DOTAGA-NPP**				
Organs	**1 h p.i.**	**4 h p.i.**	**24 h p.i.**	**48 h p.i.**	**72 h p.i.**
Blood	3.26 ± 0.57	0.36 ± 0.22	0.01 ± 0.00	0.00 ± 0.00	0.00 ± 0.00
Heart	0.91 ± 0.21	0.16 ± 0.08	0.05 ± 0.02	0.03 ± 0.01	0.02 ± 0.00
Liver	1.36 ± 0.21	1.48 ± 0.27	0.83 ± 0.16	0.51 ± 0.12	0.34 ± 0.02
Lung	2.20 ± 0.23	0.52 ± 0.16	0.10 ± 0.03	0.06 ± 0.02	0.03 ± 0.00
Pancreas	7.44 ± 1.63	1.41 ± 0.71	0.25 ± 0.07	0.18 ± 0.05	0.14 ± 0.02
Spleen	0.65 ± 0.10	0.25 ± 0.09	0.14 ± 0.03	0.11 ± 0.02	0.08 ± 0.01
Stomach	4.15 ± 0.57	1.01 ± 0.43	0.30 ±0.09	0.24 ± 0.08	0.16 ± 0.03
Intestine	0.90 ± 0.16	0.26 ± 0.07	0.18 ± 0.18	0.09 ± 0.04	0.07 ± 0.02
Adrenal	1.05 ± 0.25	0.52 ± 0.21	0.25 ± 0.12	0.22 ± 0.07	0.15 ± 0.03
Kidney	5.27 ± 1.62	5.52 ± 0.75	3.33 ± 0.41	2.13 ± 0.45	1.52 ± 0.26
Femur	0.57 ± 0.10	0.30 ± 0.15	0.08 ± 0.02	0.06 ± 0.01	0.04 ± 0.01
Muscle	0.41 ± 0.15	0.09 ± 0.04	0.04 ± 0.04	0.02 ± 0.01	0.01 ± 0.00
Tumor	23.53 ± 4.02	20.17 ± 6.15	3.82 ± 0.93	1.52 ± 0.41	0.99 ± 0.12

The results are expressed as %IA/g ± SD. Number of mice per group: 5, 8,8, 4 mice/group, respectively.

Table S5. Biodistribution data of 20 pmol (0.4-1.0 MBq) of [^{177}Lu]Lu-DOTA-PP-F11.

	**[^{177}Lu]Lu-DOTA-PP-F11**				
Organs	**1 h p.i.**	**4 h p.i.**	**24 h p.i.**	**48 h p.i.**	**72 h p.i.**
Blood	0.19 ± 0.05	0.02 ± 0.01	0.00 ± 0.00	0.00 ± 0.00	0.00 ± 0.00
Heart	0.07 ± 0.02	0.02 ± 0.00	0.01 ± 0.00	0.01 ± 0.00	0.01 ± 0.00
Liver	0.14 ± 0.02	0.09 ± 0.02	0.08 ± 0.00	0.06 ± 0.03	0.04 ± 0.00
Lung	0.26 ± 0.10	0.05 ± 0.01	0.02 ± 0.00	0.01 ± 0.00	0.01 ± 0.00
Pancreas	0.12 ± 0.01	0.05 ± 0.02	0.06 ± 0.01	0.04 ± 0.01	0.06 ± 0.01
Spleen	0.08 ± 0.01	0.04 ± 0.01	0.03 ± 0.00	0.03 ± 0.01	0.02 ± 0.00
Stomach	1.55 ± 0.53	1.45 ± 0.31	1.08 ± 0.18	0.95 ± 0.18	0.93 ± 0.23
Intestine	0.20 ± 0.08	0.13 ± 0.06	0.10 ± 0.07	0.05 ± 0.04	0.05 ± 0.03
Adrenal	0.14 ± 0.05	0.06 ± 0.01	0.05 ± 0.03	0.05 ± 0.04	0.03 ± 0.03
Kidney	3.95 ± 0.57	3.63 ± 0.64	3.21 ± 0.59	1.50 ± 0.56	1.17 ± 0.24
Femur	0.69 ± 0.27	0.21 ± 0.07	0.18 ± 0.06	0.17 ± 0.07	0.18 ± 0.07
Muscle	0.05 ± 0.01	0.02 ± 0.01	0.01 ± 0.00	0.01 ± 0.00	0.01 ± 0.00
Tumor	5.42 ± 1.42	4.66 ± 0.90	3.81 ± 0.40	1.99 ± 0.83	1.62 ± 0.20

The results are expressed as %IA/g ± SD. Number of mice per group: 4 mice/ group.

Table S6. Biodistribution data of 20 pmol (0.4-1.0 MBq) MBq of [^{177}Lu]Lu-DOTA-MGS5.

	**[^{177}Lu]Lu-DOTA-MGS5**				
Organs	**1 h p.i.**	**4 h p.i.**	**24 h p.i.**	**72 h p.i.**	**130 h p.i.**
Blood	1.28 ± 0.48	0.02 ± 0.00	0.00 ± 0.00	0.00 ± 0.00	0.00 ± 0.00
Heart	0.39 ± 0.15	0.04 ± 0.01	0.03 ± 0.01	0.02 ± 0.00	0.01 ± 0.01
Liver	0.67 ± 0.15	0.28 ± 0.05	0.21 ± 0.02	0.12 ± 0.01	0.04 ± 0.00
Lung	0.79 ± 0.24	0.07 ± 0.00	0.04 ± 0.01	0.02 ± 0.00	0.01 ± 0.00
Pancreas	0.74 ± 0.20	0.37 ± 0.16	0.33 ± 0.03	0.27 ± 0.08	0.20 ± 0.08
Spleen	0.32 ± 0.10	0.10 ± 0.01	0.09 ± 0.02	0.06 ± 0.01	0.04 ± 0.01
Stomach	10.07 ± 1.85	9.74 ± 1.97	6.22 ± 0.25	3.63 ± 0.21	1.97 ± 0.32
Intestine	0.60 ± 0.11	0.22 ± 0.03	0.22 ± 0.03	0.12 ± 0.02	0.08 ± 0.01
Adrenal	0.44 ± 0.16	0.16 ± 0.03	0.14 ± 0.03	0.09 ± 0.06	0.04 ± 0.02
Kidney	3.31 ± 0.44	2.25 ± 0.35	1.76 ± 0.28	0.88 ± 0.03	0.49 ± 0.14
Femur	0.31 ± 0.11	0.12 ± 0.02	0.07 ± 0.03	0.07 ± 0.01	0.08 ± 0.02
Muscle	0.18 ± 0.08	0.03 ± 0.01	0.03 ± 0.00	0.01 ± 0.00	0.00 ± 0.00
Tumor	18.27 ± 4.16	17.58 ± 2.48	9.36 ± 2.60	4.61 ± 0.32	0.65 ± 0.17

The results are expressed as %IA/g ± SD. Number of mice per group: 6, 4, 4, 4, 4 mice/group, respectively.

Table S7. Expected absorbed dose for the radioligands in humans

Organ	Absorbed dose per injected activity [mSv/MBq]					
	[177Lu]Lu-DOTA-PP-F11		[177Lu]Lu-DOTA-MGS5		[177Lu]Lu-DOTAGA-NPP	
	Female	Male	Female	Male	Female	Male
Small Intestine	0.017	0.014	0.016	0.013	0.016	0.013
Stomach Wall	0.002	0.002	0.181	0.138	0.018	0.014
Heart Wall	0.001	0.001	0.005	0.004	0.011	0.009
Kidneys	0.070	0.055	0.154	0.122	0.301	0.238
Liver	0.012	0.010	0.021	0.017	0.077	0.062
Lungs	0.001	0.001	0.009	0.007	0.025	0.020
Pancreas	0.002	0.002	0.033	0.026	0.075	0.061
Red Marrow	0.001	0.001	0.002	0.002	0.005	0.004
Spleen	0.001	0.001	0.010	0.008	0.016	0.013
Whole Body	0.005	0.005	0.003	0.003	0.006	0.005

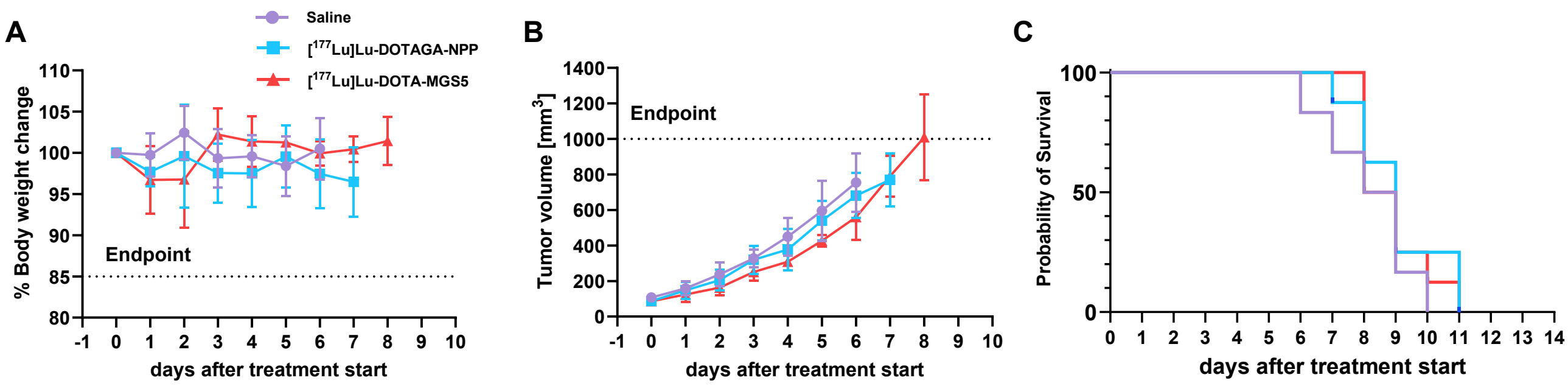

Figure S6. Therapy results obtained after treatment of A431-CCK2R+ xenograft with Saline (●), [177Lu]Lu-DOTA-NPP (■) and [177Lu]Lu-DOTA-MGS5 (▲). **A.** Mouse weight expressed as % of pre-therapy starting weight. **B.** Tumor volumes. Curves were discontinued after euthanasia of the first mouse per group. **C**. Survival rate Mouse weight expressed as % of pre-therapy starting weight.

Table S8. Biodistribution data of radiolabeled CCK2R antagonists reported in literature compared to [^{177}Lu]Lu-DOTAGA-NPP

Compound (Reference)	^{99m}Tc-CRL3 [3]	^{111}In-IP-001 [4]	^{99m}Tc-DGA1 [5]	^{111}In-GAS1 [6]	^{177}Lu-GAS1 [6]	^{111}In-GAS3 [6]	^{177}Lu-GAS3 [6]	^{177}Lu-DOTAGA-NPP (this study)
**Peptide Mass**	10 nmol	2.06 nmol	10 pmol	20-25 pmol	40 pmol	20-25 pmol	40 pmol	20 pmol
**Mouse model**	Female nu/nu	Female Balb/c	Male SCID					Female athymic Foxn1^{nu}
**Cell line**	HEK-CCK2R	A549	HEK-CCK2$_{i4sv}$R					A431-CCK2R
**Organ uptake at 4h p.i. (%IA/g)**								
**Blood**	1.7 ± 0.39	4.21 ± 0.40	0.94 ± 0.31	3.51 ± 0.56	0.99 ± 0.20	2.42 ± 0.94	3.19 ± 0.44	0.36 ± 0.22
**Kidney**	13.1 ± 2.0	6.99 ± 0.97	96.06 ± 12.01	6.06 ± 1.24	3.70 ± 0.62	9.32 ± 0.68	8.86 ± 0.67	5.59 ± 0.75
**Stomach**	0.56 ± 0.16	<1	0.75 ± 0.21	0.98 ± 0.21	0.37 ± 0.05	0.91 ± 0.39	0.64 ± 0.07	1.01 ± 0.43
**Liver**	4.1 ± 0.64	8.25 ± 2.21	1.80 ± 0.62	7.69 ± 1.00	2.21 ± 0.44	2.78 ± 0.27	2.73 ± 0.31	1.48 ± 0.27
**Pancreas**	0.29 ± 0.19	2.03 ± 0.16	0.38 ± 0.09	1.49 ± 0.19	0.56 ± 0.11	0.86 ± 0.23	1.00 ± 0.13	1.41 ± 0.71
**Tumor**	12.0 ± 2.0	2.36 ± 0.26	31.63 ± 4.59	17.79 ± 1.73	8.74 ± 1.60	19.83 ± 1.35	20.88 ± 1.20	20.17 ± 6.15
**Tumor-to-organ ratios**								
**Blood**	7.1	0.6	33.7	5.1	8.8	8.2	6.6	56.0
**Kidney**	0.9	0.3	0.3	2.9	2.4	2.1	2.4	3.6
**Stomach**	21.4	-	42.2	18.2	23.6	21.8	32.6	20.0
**Liver**	2.9	0.3	17.6	2.3	4.0	7.1	7.7	13.6

References

1. Novak D. Novi radiooznačeni antagonisti holecistokinin-2/gastrinskega receptorja : doktorska disertacija [Doctoral dissertation]. Ljubljana: Faculty of Pharmacy; 2021.
2. Novak D, Tomasic T, Kroselj M, Javornik U, Plavec J, Anderluh M, et al. Radiolabelled CCK(2) R Antagonists Containing PEG Linkers: Design, Synthesis and Evaluation. ChemMedChem. 2021; 16: 155-63.
3. Wayua C, Low PS. Evaluation of a nonpeptidic ligand for imaging of cholecystokinin 2 receptor-expressing cancers. J Nucl Med. 2015; 56: 113-9.
4. Verona M, Rubagotti S, Croci S, Sarpaki S, Borgna F, Tosato M, et al. Preliminary Study of a 1,5-Benzodiazepine-Derivative Labelled with Indium-111 for CCK-2 Receptor Targeting. Molecules. 2021; 26.
5. Kaloudi A, Kanellopoulos P, Radolf T, Chepurny OG, Rouchota M, Loudos G, et al. [(99m)Tc]Tc-DGA1, a Promising CCK(2)R-Antagonist-Based Tracer for Tumor Diagnosis with Single-Photon Emission Computed Tomography. Mol Pharm. 2020; 17: 3116-28.
6. Nock BA, Kanellopoulos P, Chepurny OG, Rouchota M, Loudos G, Holz GG, et al. Nonpeptidic Z360-Analogs Tagged with Trivalent Radiometals as Anti-CCK(2)R Cancer Theranostic Agents: A Preclinical Study. Pharmaceutics. 2022; 14.
